# pH Effects in a Model Electrocatalytic Reaction Disentangled

**DOI:** 10.1021/jacsau.2c00662

**Published:** 2023-03-01

**Authors:** Xinwei Zhu, Jun Huang, Michael Eikerling

**Affiliations:** † Theory and Computation of Energy Materials (IEK-13), Institute of Energy and Climate Research, Forschungszentrum Jülich GmbH, 52425 Jülich, Germany; ‡ Chair of Theory and Computation of Energy Materials, Faculty of Georesources and Materials Engineering, RWTH Aachen University, 52062 Aachen, Germany

**Keywords:** pH effects, local reaction environment, electric double layer, mass transport, surface charging effects, formic acid oxidation reaction

## Abstract

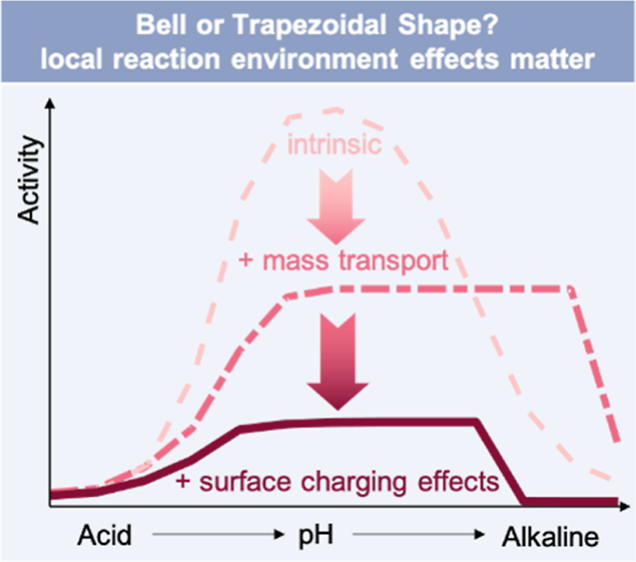

Varying the solution pH not only changes the reactant concentrations in bulk solution but also the local reaction environment (LRE) that is shaped furthermore by macroscopic mass transport and microscopic electric double layer (EDL) effects. Understanding ubiquitous pH effects in electrocatalysis requires disentangling these interwoven factors, which is a difficult, if not impossible, task without physical modeling. Herein, we demonstrate how a hierarchical model that integrates microkinetics, double-layer charging, and macroscopic mass transport can help understand pH effects of the formic acid oxidation reaction (FAOR). In terms of the relation between the peak activity and the solution pH, intrinsic pH effects without consideration of changes in the LRE would lead to a bell-shaped curve with a peak at pH = 6. Adding only macroscopic mass transport, we can already reproduce qualitatively the experimentally observed trapezoidal shape with a plateau between pH 5 and 10 in perchlorate and sulfate solutions. A quantitative agreement with experimental data requires consideration of EDL effects beyond Frumkin correlations. Specifically, the peculiar nonmonotonic surface charging relation affects the free energies of adsorbed intermediates. We further discuss pH effects of FAOR in phosphate and chloride-containing solutions, for which anion adsorption becomes important. This study underpins the importance of a full consideration of multiple interrelated factors for the interpretation of pH effects in electrocatalysis.

## Introduction

Electrocatalysis, which refers to heterogeneous catalysis of electrochemical reactions that occur at the electrode–electrolyte interface, is the key discipline to enable a sustainable and environmentally benign global energy infrastructure. A quintessential aspect of electrocatalysis is that the activity of a particular reaction can vary over several orders of magnitude if different electrode materials or atomic surface configurations are used.^1−3^ In the past decades, remarkable progress in understanding the role of the electrode material has paved the way toward rational designs of electrocatalysts.^1−8^ The role of other medium involved in electrocatalysis, viz. the electrolyte, is however much less understood. Controversy exists over how the type and concentration of cations modulate the reaction activity,^9−12^ and why variations in solution pH cause activity differences of several orders of magnitude for specific reactions.^13−18^

Various explanations of pH effects in electrocatalytic reactions have been given, including shifts of bulk proton concentration and electrode potential on the standard hydrogen electrode (SHE) scale,^18,19^ changes in proton donor or oxidant,^14,20,21^ and modulations on possible solution-phase reactions.^22−24^ In addition to these overall properties, local properties in the electric double layer (EDL), including local potential, concentration, and electric field, can also change with the solution pH. Recent years have witnessed growing attention to these local reaction environment (LRE) effects. For instance, macroscopic mass transport induces departure of the local pH from the bulk pH during the formic acid oxidation reaction (FAOR).^25,26^ Additionally, the local reactant concentration, which is lower than the bulk concentration due to the macroscopic mass transport, is a key factor to understand pH effects on the hydrogen evolution/oxidation reaction (HER/HOR)^14,27^ and the CO_2_ electrochemical reduction reaction (CO_2_ER).^28,29^ It has also been demonstrated that the electric field in the EDL impacts free energies of intermediates in the oxygen reduction reaction^30,31^ and the CO electrochemical reduction.^9,32^ Lastly, the role of the reorganization of interfacial water affected by the electric field is highlighted in the HER.^33,34^ These studies have shown that apparent pH effects are the consequence of multiple interacting factors. Any endeavor toward a comprehensive understanding should be aware of their interplay and find a proper way to disentangle them, which is highly nontrivial even for simple model reactions, such as HER and FAOR.^34,35^ Theory and modeling are indispensable to this end.^1,2,15^

In this work, we aim to disentangle the aforementioned multiple factors that affect the FAOR at single crystals. Marked pH effects have been reported by several independent groups for this model system, but the explanations given have remained controversial. The controversy on reproducible experimental phenomena in the simplest model system just indicates the complexity arising from multiple interacting factors, as well as the lack of a reliable approach to handle the complexity. We carefully analyze experimental data using a set of models that gradually climb the ladder of complexity, in order to avoid overfitting of experimental data. Our incremental analysis underlines the importance of considering LRE in understanding experimental pH effects. Specifically, we find that a quantitative analysis of experiments requires taking into account EDL effects that go beyond Frumkin corrections but were not considered in previous studies.

## Model Development

We model the oxidation of formic acid for a system consisting of a rotating Pt(111) disk electrode (RDE) in 0.5 M perchlorate solutions under ambient conditions (25 °C, 101.3 kPa). The pH range from 0 to 12 is considered. The model consists of a microkinetic submodel that determines the adsorbate coverages and current densities and a modified Poisson–Nernst–Planck (PNP) submodel that determines the LRE, namely, the distributions of potential and species concentrations at or near the reaction plane (RP).

### Microkinetics

The reaction mechanism used in this model involves three adsorbates, HCOO_b_, HCOO_m_, and OH_ad_^36^
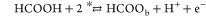
Step 1
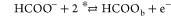
Step 2
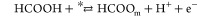
Step 3
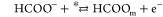
Step 4
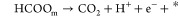
Step 5
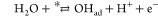
Step 6
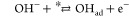
Step 7Bridge-bonded formate HCOO_b_ was detected on Pt electrodes by surface-enhanced IR absorption spectroscopy.^37^ However, HCOO_b_ is unlikely to be the main active intermediate because density functional theory (DFT) calculations have shown that its oxidation barrier is too high (∼1.1 eV).^38,39^*In situ* IR studies have shown that HCOO_b_ is a site-blocking spectator.^40,41^ Therefore, the oxidation step of HCOO_b_ should be negligible. It has then been speculated that there must be other active intermediates, which, due to their short lifetime and/or low coverage, were not detected in spectroscopic measurements.^41^ DFT calculations have proposed two candidates, COOH_ad_^42^ and C–H down monodentate formate HCOO_m_.^43,44^ Following studies on the pH effects of FAOR,^24,45−47^ we consider HCOO_m_ as the main active intermediate. Herein, we have negligible adsorbed CO_ad_ because CO_ad_ is considered to be absent on defect-free Pt(111).^48^ In addition, cyclic voltammetry data in the negative scan during which CO_ad_ is oxidized are used for the analysis.^45,49,50^ In addition to the main reactions, this model also considers water oxidation at high potentials that forms site-blocking OH_ad_.

For the case of phosphate solutions, the FAOR is inhibited when pH > 4.^45,51^ This is the pH range in which HPO_4_^2–^ appears. Therefore, we consider the adsorption of HPO_4_^2–^
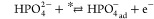
Step 8

For the case of chloride-containing solutions, anion adsorption is accounted for *via*
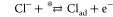
Step 9

Based on the postulated reaction mechanism, rate equations for the changes of adsorbate coverages, which are zero at steady state, can be formulated
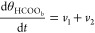
1
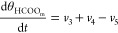
2
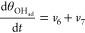
3
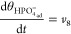
4
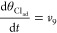
5where , , , , and  are coverages and *v*_*i*_ is the reaction rate of step *i*
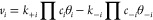
6where *c*_*i*_ and θ_i_, *c*_–*i*_, and θ_–*i*_ are species concentrations and intermediate coverages involved in forward and backward reactions of step *i*. The rate constants *k*_+*i*_ and *k*_–*i*_ are obtained from transition-state theory
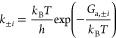
7with *k*_B_ being the Boltzmann constant, *T* the temperature, and *h* the Planck constant. Activation energies, *G*_a,±*i*_, under standard conditions (298 K, 1 bar pressure, pH = 0) are determined using the Brønsted–Evans–Polanyi (BEP) relation^52^
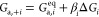
8
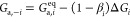
9where *G*_a,*i*_^eq^ is the activation energy of step *i* under standard equilibrium conditions, β_*i*_ is the transfer coefficient, and Δ*G*_*i*_ is the reaction Gibbs free energy, which shifts with potential according to
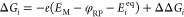
10Here, *e* is the elementary charge, *E*_M_ is the applied potential relative to the SHE, and φ_RP_ is the potential at the RP, which needs to be determined with the PNP submodel. *E*_*i*_^eq^ is the equilibrium potential of step *i* and is calculated using the Nernst equation
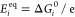
11with Δ*G*_*i*_^0^ being the reaction free energy of step *i* at the standard state, which can be determined with the aid of DFT. ΔΔ*G*_*i*_ represents the variations in the Gibbs free energy caused by lateral interactions between adsorbates and surface charging effects.

, , , , and  are obtained by solving eqs 1–5. The partial current densities are given by
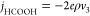
12
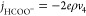
13
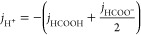
14
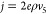
15with ρ the number density of active sites at Pt(111) and *e*ρ = 2.41 C/m^2^ in this model.^53^ The details and parameters of the microkinetic model can be found in the Supporting Information.

### Local Reaction Environment

**Figure 1:**
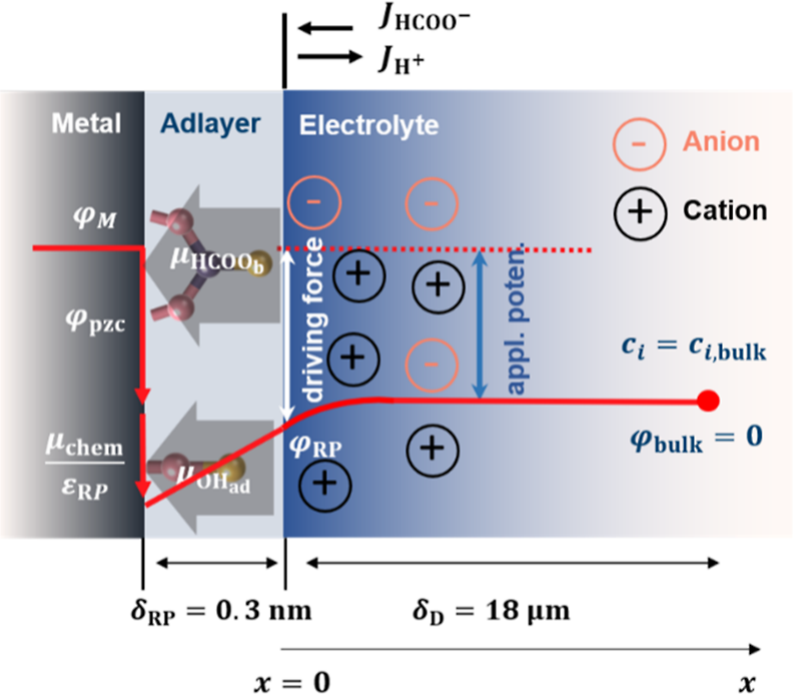
Schematic illustration of the model system. Two zones at the electrode–electrolyte surface are distinguished: the adlayer and the boundary layer in the electrolyte. The RP is located at the boundary between them, where the fluxes are indicated. The solid red line is a schematic potential profile for the case of negative surface charge, with φ_M_ denoting the applied metal potential and φ_pzc_ the potential of zero charge. μ_chem_ is the dipole moment associated with chemisorbed HCOO_b_ and OH_ad_. The driving force for interface reactions is defined as the difference between the applied potential φ_M_ and the potential at the RP φ_RP_. δ_RP_ and δ_D_ are the thicknesses of adlayer and diffusion layer, respectively.

The model system is shown in Figure 1. We consider an adlayer between the electrode and the electrolyte, in which the intermediates are adsorbed, and the potential distribution across the adlayer is considered to be linear. The RP is located at the outer surface of the adlayer (*x* = 0), which is also the position of the closest ion. Outside the RP resides the diffusion layer, whose thickness is determined as^54^
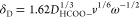
16where  is the diffusion coefficient of HCOO^–^, *v* is the kinematic viscosity of the solution phase, and ω is the rotation speed of the RDE. We use  to calculate the diffusion layer thickness since HCOO^–^ is the main reactant.

The modified Nernst–Planck equation, which takes into account steric effects,^55,56^ is used to model mass transport of HCOOH, HCOO^–^, OH^–^, ClO_4_^–^, H^+^, Na^+^, H_3_PO_4_, H_2_PO_4_^–^, HPO_4_^2–^, and PO_4_^3–^
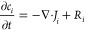
17
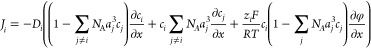
18where *c*_*i*_ is the concentration, *R*_*i*_ is the source term due to homogeneous reactions (cf. Supporting Information Note S2), *J*_*i*_ is the flux, *D*_*i*_ is the diffusion coefficient, *N*_A_ is the Avogadro constant, *a*_*j*_ is the effective diameter, *z*_*i*_ is the charge number, *F* is the Faraday constant, *R* is the ideal gas constant, and φ is the local electric potential.

The Nernst–Planck equation is complemented by the Poisson equation
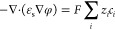
19with ε_s_ being the permittivity of the electrolyte.

To solve the PNP equations, boundary conditions are needed. At the right side of the domain shown in Figure 1 (*x* = δ_D_), concentrations are equal to bulk concentrations, *i.e.*, *c*_*i*_ = *c*_*i*,bulk_. The potential at this position is taken as the reference potential, φ_bulk_ = 0. The left side (*x* = 0) corresponds to the RP. The fluxes at this position are given by
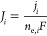
20where *j*_*i*_ is the partial current density that consumes (*j*_*i*_ < 0) or generates (*j*_*i*_ > 0) species *i*, as described in eqs 12–14, and *n*_e,*i*_ is the number of electrons involved in the reaction. We have , , and *J*_*i*_ = 0 for other species. The boundary condition for the potential is given by a Robin-type boundary condition^55,57^
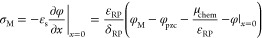
21where σ_M_ is the free surface charge density, ε_RP_ is the permittivity of the adlayer, and μ_chem_ is the chemisorption-induced surface dipole moment^58,59^
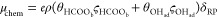
22with  and  being the net charge numbers of HCOO_b_ and OH_ad_. The dipole moment of HCOO_m_ has been neglected due to the negligible coverage of this adsorbate.

All the parameters used in the PNP model are summarized in the Supporting Information. The model was solved using COMSOL Multiphysics.

## Results

In this work, we mainly focus on the relation between the peak current density and the pH value, namely, the *j*_p_–pH relation, which is the most discussed relation in studies of the pH effects in FAOR.^24,45^ Our analysis begins with exclusive consideration of the microkinetics. We then add mass-transport effects and surface charging effects in sequential steps. This approach allows disentangling different factors controlling the overall pH effects.

### Intrinsic Microkinetics

**Figure 2:**
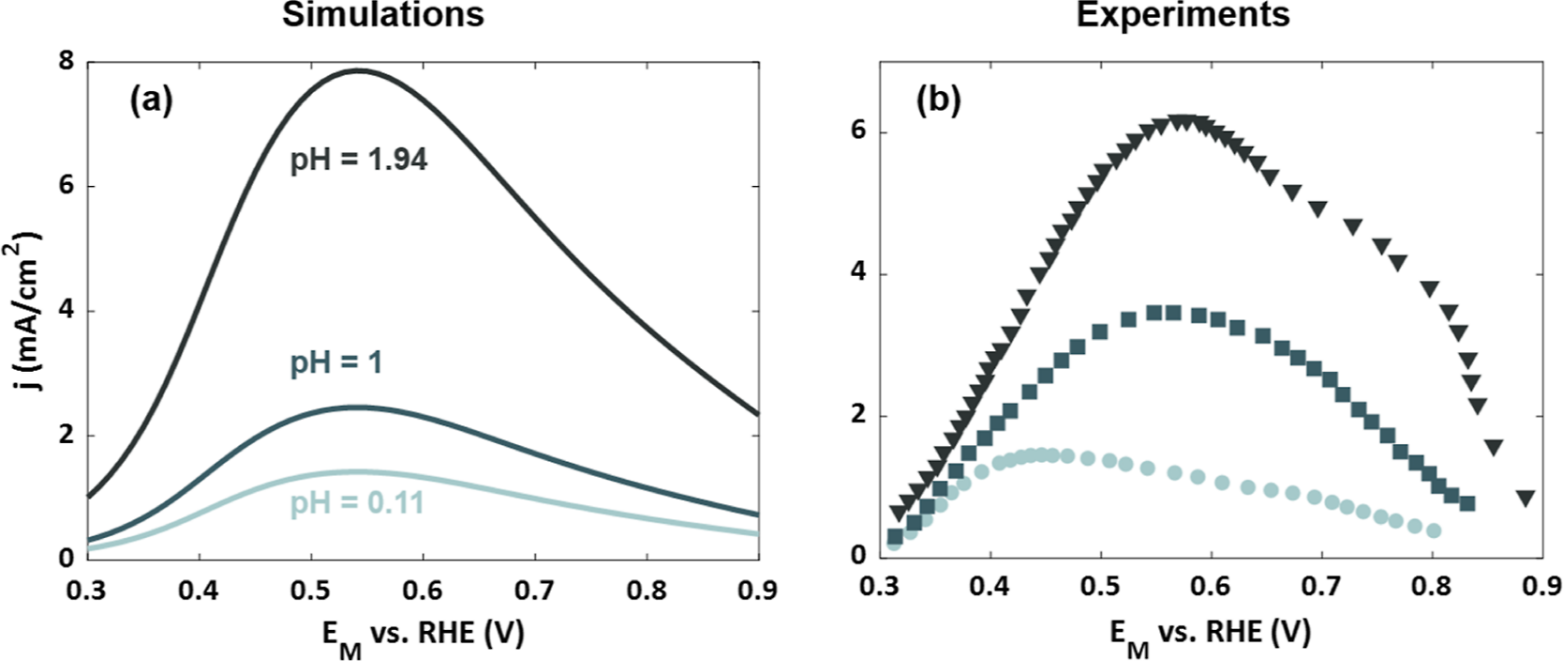
Comparison of simulated and experimental *j*–*E*_M_ curves at pH = 0.11 (1 M HClO_4_), pH = 1 (0.1 M HClO_4_), and pH = 1.94 (0.01 M HClO_4_ + 0.09 M NaClO_4_). The concentration of formic acid is 0.1 M. Experimental data are taken from ref (60).

We define the intrinsic microkinetics as reaction kinetics without accounting for effects of the LRE, which means that we consider *c*_*i*_ = *c*_*i*,bulk_, φ_RP_ = 0, and σ_M_ = 0. We start with simulating the polarization curves, namely, the *j*–*E*_M_ curves, at different pH values using the parameters in Supporting Information Tables S1 and S2. Figure 2 compares the simulations and experiments. The model captures key features of the experimental data, namely, the *j*–*E*_M_ curve presents a bell shape, and the activity increases with pH. However, there are quantitative differences between simulations and experiments. There could be two reasons. On the one hand, the LRE is not considered, and on the other hand, there are also noticeable discrepancies between experiments (Figure S2).

**Figure 3:**
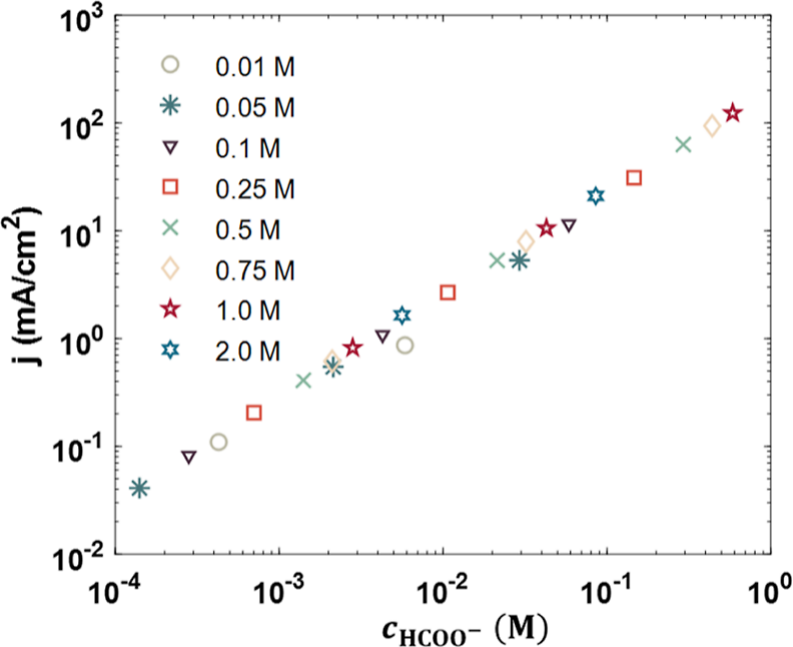
Simulated current densities at 0.13 V *vs* SHE as a function of the formate concentration in solutions with pH 1.2, 2.4, and 3.9. The total concentration of formic acid considered is between 0.01 and 2 M. The different symbols refer to the total formic acid concentrations.

Recently, Herrero *et al.* reported that the reaction order for formate is 1.^47^ To further validate the purported microkinetic model, we calculate the reaction order with respect to the formate concentration in solutions with pH 1.2, 2.4, and 3.9 at 0.13 V *vs* SHE and for concentrations of formic acid between 0.01 and 2 M. The results in Figure 3 confirm that the reaction order is nearly 1.

**Figure 4:**
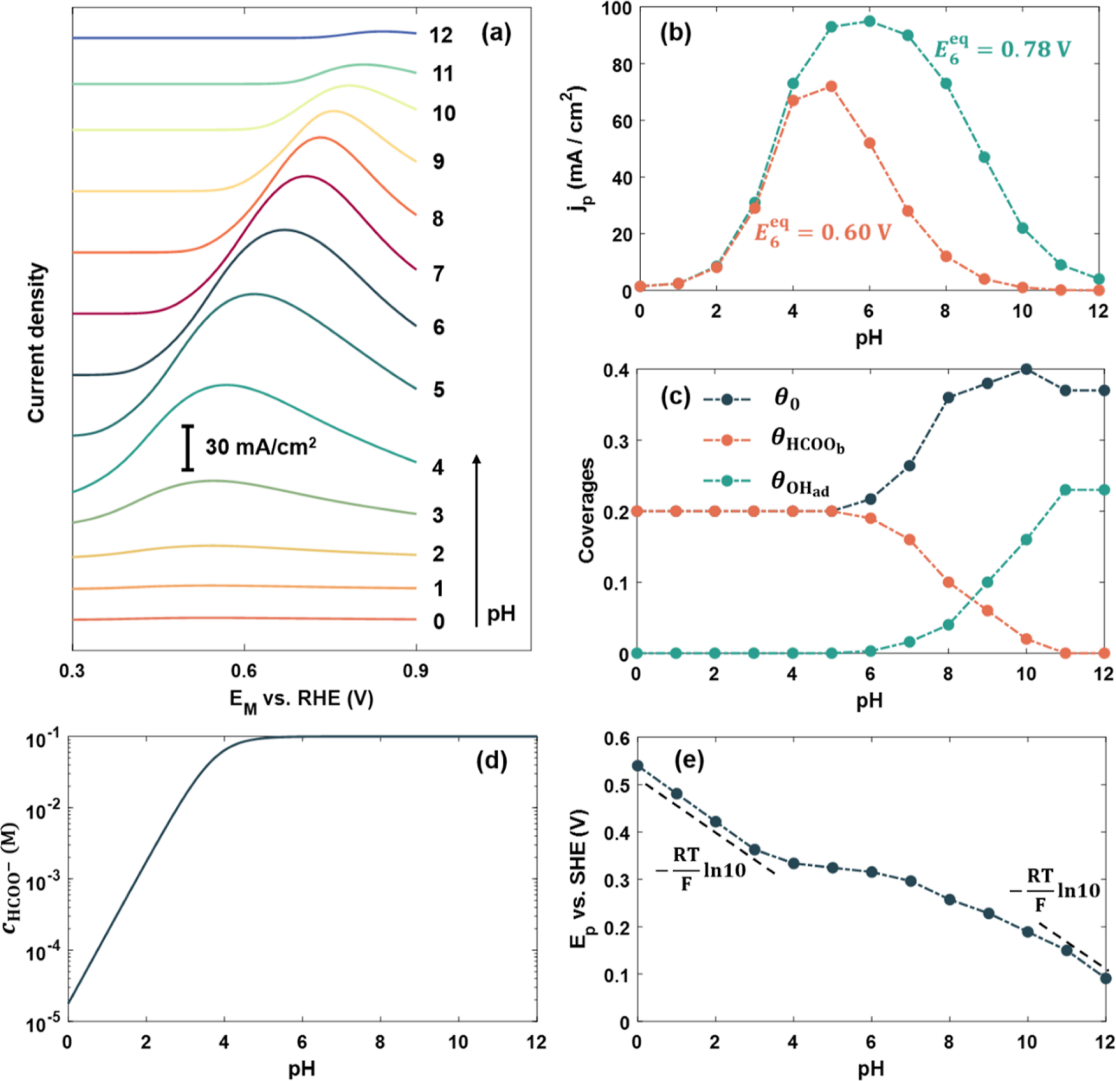
(a) Simulated *j*–*E*_M_ curves in the pH range from 0 to 12. (b) Simulated *j*_p_–pH relations for *E*_6_^eq^ = 0.78 V (used in this work) and *E*_6_^eq^ = 0.60 V (used in refs (24) and (61)). (c) Coverages of free sites, HCOO_b_ and OH_ad_ at peak potentials. (d) Bulk concentration of HCOO^–^ at different pH values. (e) Peak potentials on the SHE scale, *E*_p,SHE_.

We then extend the model to a wide pH range. Figure 4a shows the simulated *j*–*E*_M_ curves at pH from 0 to 12. The *j*_p_–pH relation, obtained from Figure 4a, is shown in Figure 4b. We show that the *j*_p_–pH relation presents a bell shape, with the peak located at pH = 6, not the p*K*_a_ of formic acid as previously thought.^24,61^ In addition, the peak current density remains almost constant between pH 5 and 7, with a difference below 5%. We notice that in the work of Joo *et al.*(24) and Zhang *et al.*,^61^*E*_6_^eq^ = 0.6 V (SHE) is used to obtain the bell-shaped *j*_p_–pH relation with the peak near the p*K*_a_ of formic acid. However, 0.6 V is much lower than the common equilibrium potential of OH_ad_ adsorption at Pt(111), which is about 0.8 V,^53,62^ resulting in an overestimation of the site-blocking effect of OH_ad_. The comparison of  for *E*_6_^eq^ = 0.78 V and *E*_6_^eq^ = 0.6 V is provided in Figure S3.

To understand the physical origins behind the bell-shaped *j*_p_–pH relation, we give an analytical solution of the peak potential *E*_p_, which is defined as the potential where the peak current density lies at a specific pH. However, since there are three adsorbates, HCOO_b_, HCOO_m_ and OH_ad_, and HCOO_b_ occupies two sites, the system is highly nonlinear (see eqs S1–S7), and it cannot be solved analytically. Instead of solving *E*_p_ directly, we solve  and  at *E*_p_, which is very helpful for the analysis of the *j*_p_–pH relation. We neglect lateral interactions between adsorbates for the moment to obtain the analytical solution. The validity of this assumption will be discussed later.

Considering that HCOO^–^ is the main reactant and the adsorption of HCOO_m_ is the rate-determining step (see Supporting Information Note S1), the turnover frequency (TOF) can be written as
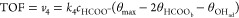
23The adsorption isotherms of HCOO_b_ and OH_ad_ are
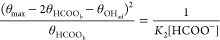
24
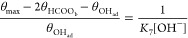
25with  being the equilibrium constant of step *i*.

At the peak potential, we have . Differentiating eq 23 with respect to *E*_M_, we obtain
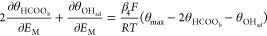
26Differentiating eqs 24 and 25 with respect to *E*_M_, we then find
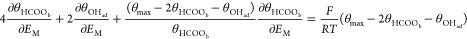
27
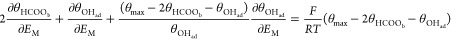
28Solving eqs 26, 27 and 28 together, we get
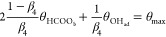
29where θ_max_ = 0.6 and β_4_ = 0.4 (see Supporting Information Note S1) in this work. In the case where the OH_ad_ adsorption can be negligible, for example at acidic pH,^63^ we have
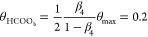
30The peak potential only exists when β_4_ < 0.5 since  (HCOO_b_ occupies two sites). The sensitivity of β_4_ in the *j*–*E*_M_ curve at pH = 1 is shown in Figure S4.

In the case where the HCOO_b_ adsorption can be negligible, we have
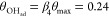
31The peak potential always exists in this case. Numerical calculations considering the lateral interactions do not alter the above conclusion, as shown in Figure S5.

The simulated peak potentials lie at  when pH < 5, in which case the OH_ad_ adsorption is negligible, as shown in Figure 4c. This is consistent with the analytical expression of eq 30. Specifically,  shown in Figure 4d increases approximately 10 times if pH increases by 1 when pH < 3 due to the dissociation equilibrium of formic acid. Therefore, the peak potential *E*_p,SHE_ must decrease by  to maintain  according to eq 24, as shown in Figure 4e. The peak current density will accordingly increase by  times since θ_0_ is constant. When 5 < pH < 10,  is constant, *E*_p,SHE_ decreases, and θ_0_ increases, resulting in a nonmonotonic *j*_p_–pH relation. The adsorption of OH_ad_ is dominant, and the adsorption of HCOO_b_ is negligible when 11 < pH < 12. Therefore, *E*_p,SHE_ decreases by  to keep  according to eq 25, leading to a decrease in activity.

### Mass-Transport Effects

**Figure 5:**
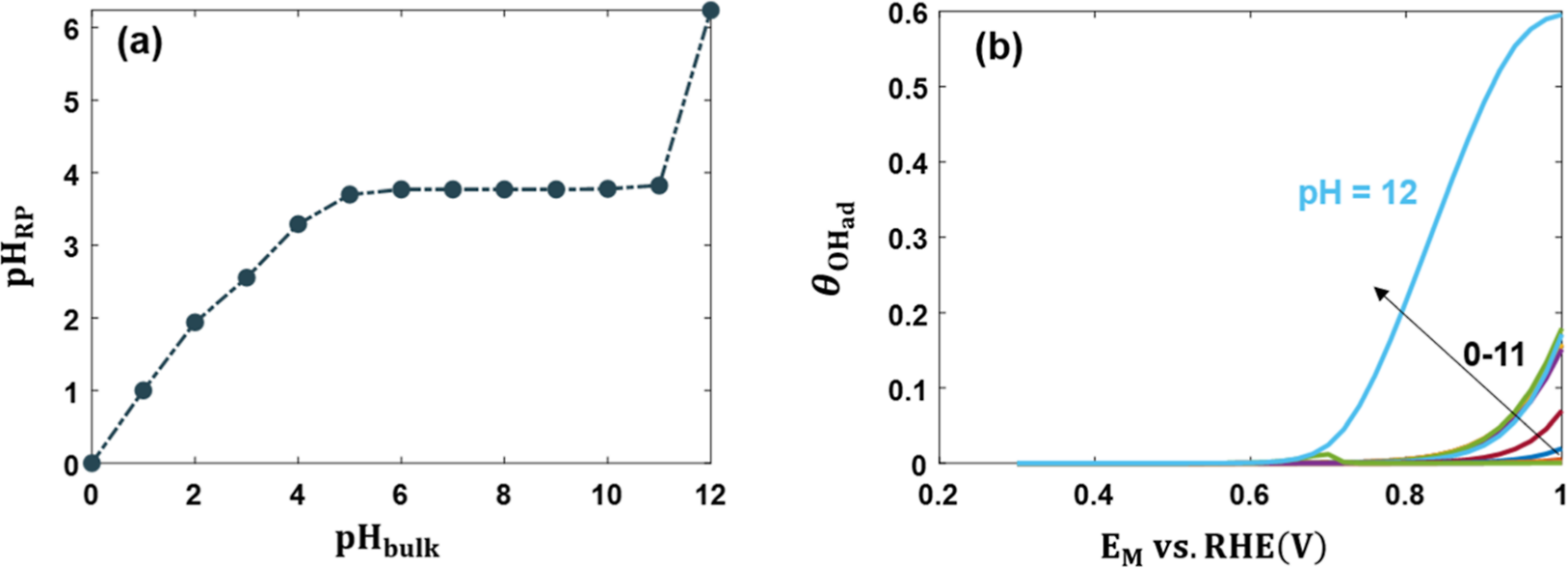
(a) Comparison of the surface pH and the bulk pH at peak potentials. (b) Adsorption isotherm of OH_ad_ in the pH range of 0 and 12.

In this section, we add mass transport of species into the play, while the effects of surface charging are not yet considered, *i.e*., φ_RP_ = 0 and σ_M_ = 0. Since the FAOR generates protons, the solution phase in close proximity to the electrode surface should be more acidic than the solution phase in the bulk. Figure 5a shows the simulated behavior of the pH at the RP, pH_RP_, at the peak potential as a function of the bulk pH. pH_RP_ deviates strongly from the bulk pH for pH_bulk_ > 4. In the range of 5 < pH_bulk_ < 11, pH_RP_ remains constant at pH_RP_ ≈ 4, which is consistent with previous simulations.^25^ Due to the inhibited growth of pH_RP_ with pH_bulk_, OH_ad_ adsorption is suppressed, as shown in Figure 5b. For comparison, the isotherm of OH_ad_ adsorption without accounting for mass-transport effects is provided in Figure S6.

**Figure 6:**
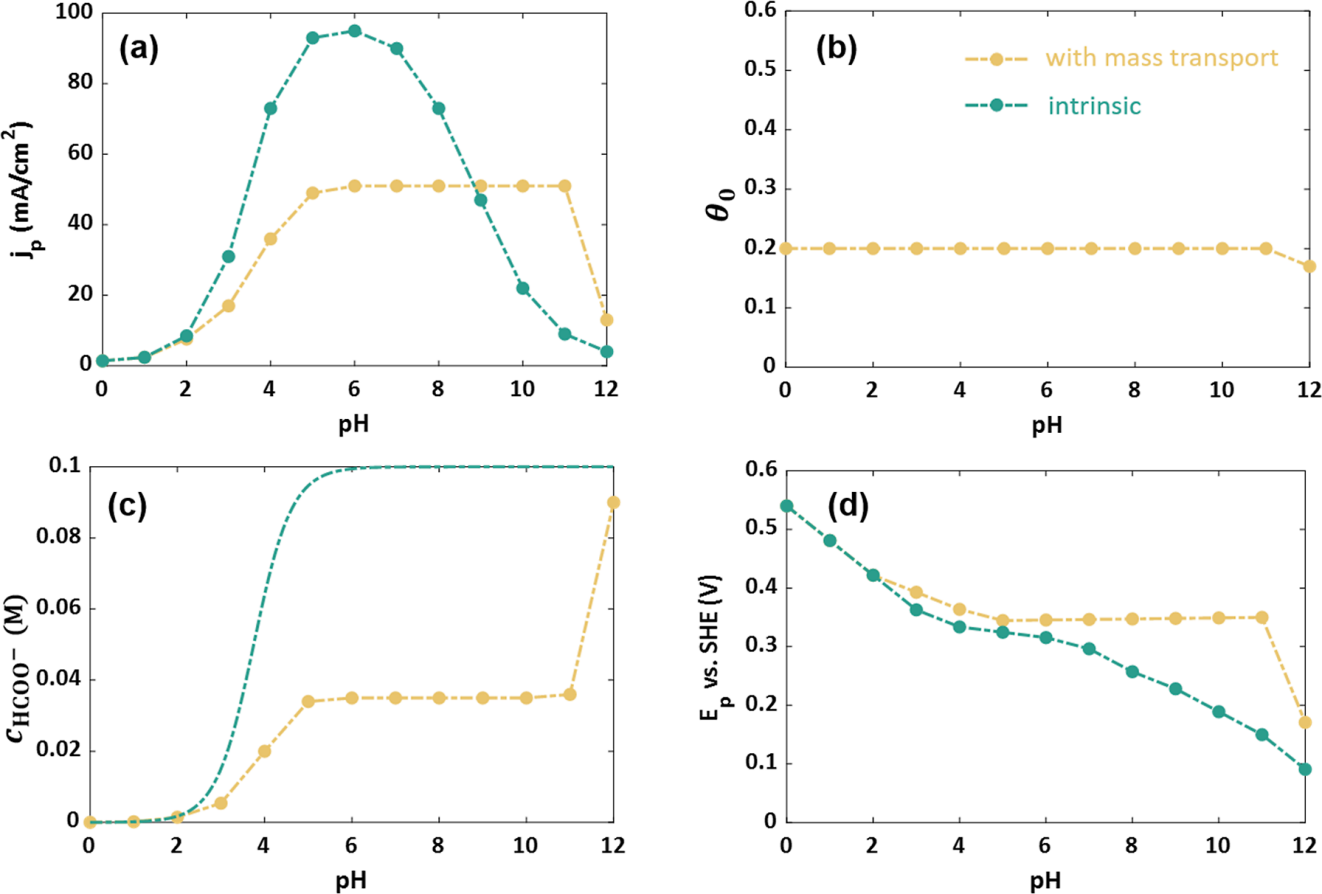
(a) Comparison of the *j*_p_–pH relation considering only intrinsic microkinetics and the *j*_p_–pH relation corrected by mass-transport effects. (b) Coverage of free sites at peak potentials. (c) Comparison of the bulk and the surface concentrations of HCOO^–^. (d) Comparison of the intrinsic microkinetics controlled peak potentials and the mass-transport-corrected peak potentials.

Under the influence of mass-transport effects, the *j*_p_–pH relation transforms from a bell shape to a trapezoidal shape, with a plateau between pH 5 and 11, as shown in Figure 6a. In this pH range, OH_ad_ adsorption is negligible, Figure 5b. Therefore, the peak potential remains at , as shown in Figure 6b. We show θ_0_ here since . The isotherm of HCOO_b_ adsorption given in Figure S7 is consistent with the trend in experimental data in refs (26) and (64). Since  at the RP is constant in this pH range, as seen in Figure 6c, *E*_p,SHE_ must be fixed to maintain , Figure 6d. The activity therefore remains constant according to eq 23.

In addition, mass transport exerts two competing effects: decreasing the reactant concentration  at the RP, Figure 6c, and increasing the driving force, Figure 6d, by suppressing the OH_ad_ adsorption. When pH < 9, the effect of decreasing  dominates, leading to a decrease in activity. When pH > 9, the effect of increasing the driving force dominates, which promotes the activity.

**Figure 7:**
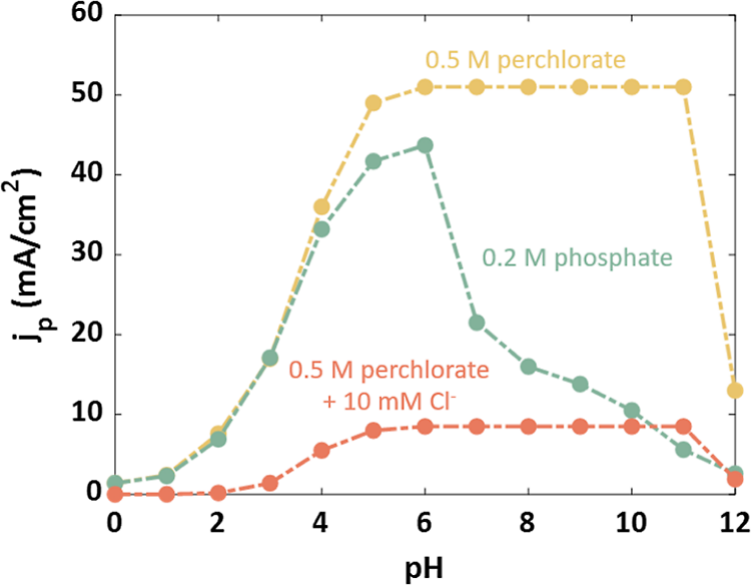
Mass-transport-corrected *j*_p_–pH relations in 0.5 M perchlorate, 0.2 M phosphate, and 0.5 M perchlorate + 10 mM chloride solutions.

It has been proposed that the *j*_p_–pH relation in phosphate solution is intrinsic because of the buffering capacity of the electrolyte.^25^ We calculate the surface pH and the mass transport corrected *j*_p_–pH relation in 0.2 M phosphate solution without considering the specific adsorption of phosphate anions. The results are shown in Figure S8. Compared with the case in perchlorate solution, the mass-transport effects are weakened due to the buffer reactions (Steps S3–S5). Correspondingly, the activity in phosphate solution is higher than that in perchlorate solution when pH < 9, contrary to the experimental observation that the activity in phosphate solution is much lower than that in perchlorate solution.^24,45,49,65^ This discrepancy implies that the strong anion adsorption in phosphate solution plays a more important role than its buffering capacity. In a further step, we include the specific adsorption of phosphate anions (Step 8) in the microkinetics. The result in Figure 7 shows that the *j*_p_–pH relation in phosphate solution is bell-shaped with the peak at pH = 6. This trend is consistent with experimental data on Pt(111) in ref (51). However, on polycrystalline Pt, the peak is located at pH = 4.^24,45^ This may be due to the fact that adsorption of phosphate anions on polycrystalline Pt is different from that on Pt(111).^66^ In addition, Figure 7 shows that the site-blocking effect of adsorbed phosphate is negligible when pH < 4 and becomes significant when pH > 6, in agreement with the concentration profile of HPO_4_^2–^*versus* pH. Unlike HPO_4_^2–^, whose concentration is pH-dependent, added Cl^–^ has a pH-independent concentration. Therefore, the site-blocking effect of Cl^–^ is significant at any pH. The simulated *j*_p_–pH relation in perchlorate solution containing 10 mM Cl^–^ captures the experimental trend in ref (45).

### Surface Charging Effects

So far, we have explained the physical origin of the experimentally observed trapezoidal-shaped *j*_p_–pH relation in perchlorate solution by combining microkinetic modeling with mass-transport effects in the previous section.^45^ However, we notice that the simulated current density in the plateau region is about three times higher than the experimental data..^43,45^ One may assume that this is due to the underestimation of mass-transport effects. Therefore, we examine the sensitivity of the diffusion layer thickness. As shown in Figure S9, the simulated current density in the flat region is still about two times higher than the experiments even we increase δ_D_ to 30 μm, which corresponds to ω = 350 rpm, while the experiments are usually conducted in ω > 1000 rpm.^45^ Analytically, we have found that *j*_p_ increases by about four times if pH increases by 1 when pH < 3, which means that *j*_p_ = 16 mA/cm^2^ at pH = 3 if *j*_p_ = 1 mA/cm^2^ at pH = 1, while experiments give *j*_p_ ≈ 3 mA/cm^2^ at pH = 1.^60,67^ Combined, there should be other factors that affect the *j*_p_–pH relation, and we are about to show that surface charging effects are very likely responsible for this. The surface charging effects considered in this work contain two aspects, *i.e*., the Frumkin effects that the reactant concentration and potential at the RP are controlled not only by the mass-transport effects but also by the electrode surface charge density, and the Gibbs free energies of intermediates that depend on it.

**Figure 8:**
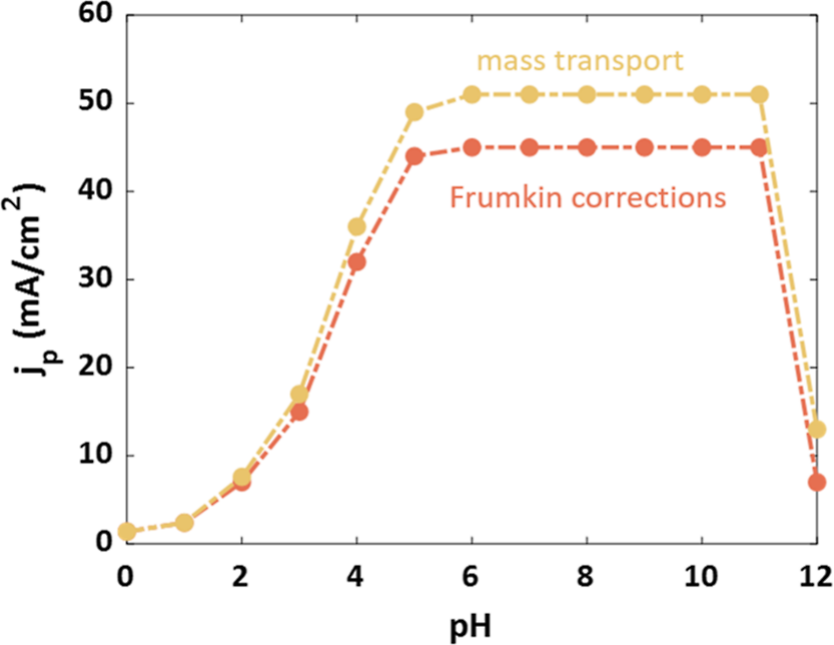
Comparison of the *j*_p_–pH relation corrected by only mass-transport effects and the *j*_p_–pH relation corrected by Frumkin corrections in perchlorate solution.

We first consider the Frumkin corrections. The simulated *j*_p_–pH relation is shown in Figure 8. Compared with the case of that includes mass-transport effects only, the inclusion of Frumkin corrections does not significantly change the trend but only slightly reduces the activity. This is because the negative surface charge has two competing impacts (as shown in Figure 1): it increases the driving force and decreases the surface concentration of HCOO^–^,  (opposite for positive surface charge). The two competing effects more or less cancel each other off in this case. This balance is not sensitive to the key parameters of the EDL, ε_RP_ and δ_RP_, as shown in Figure S10. Comparison of the *j*_p_–pH relations corrected by only mass-transport effects and the *j*_p_–pH relations corrected by Frumkin corrections in phosphate and chloride-containing solutions is shown in Figure S11.

Recent theoretical studies have shown that the formation energies of adsorbates are dependent on the surface charge.^57,68^ Moreover, it has been proposed that the positive surface charge is more favorable for formate adsorption.^44,69^ To include such surface charge effects beyond Frumkin corrections, we assume that variations in the Gibbs free energies of adsorption of HCOO_b_ and HCOO_m_ are linearly correlated with the surface charge density
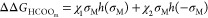
32
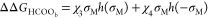
33where  for σ_M_ > 0 and  elsewise, , . eqs 32 and 33 imply that a positive surface charge density supports the formate adsorption while a negative surface charge density suppresses it. The suppression effect is more pronounced for HCOO_m_.

**Figure 9:**
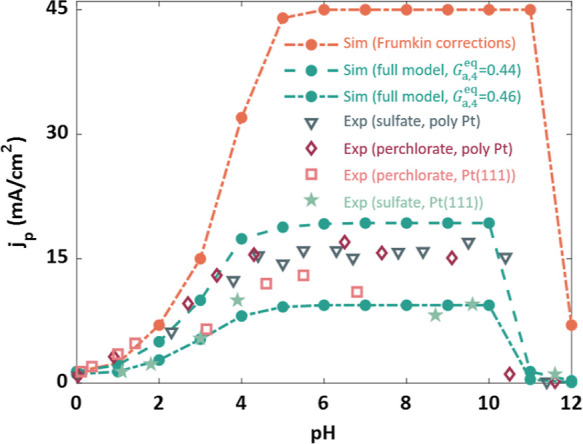
Comparison of the simulated *j*_p_–pH relations and the experimental *j*_p_–pH relations in perchlorate and sulfate solutions. The concentration of formic acid is 0.1 M. The sensitivity of *G*_a,4_^eq^ is examined to cover the experimental fluctuations. Experimental data for polycrystalline Pt are taken from ref (45), for Pt(111) in perchlorate solution from ref (43), and for Pt(111) in sulfate solution from ref (25).

The surface charge becomes more negative with increasing pH (Figure S12) since φ_pzc_ on the reversible hydrogen electrode (RHE) scale increases 0.059 with pH increasing by 1. Due to the negative shift of the surface charge density, the adsorption of HCOO_m_ is suppressed, and the activity is reduced correspondingly. As shown in Figure 9, we achieve quantitative agreement with experiments after taking into account the surface charging effect on the free energies of intermediates. In addition, the *j*_p_–pH relation is sensitive to the activation barrier of the key reaction step, *G*_a,4_^eq^. A range of only 0.02 eV for this parameter can cover the experimental fluctuations. This implies that kinetic factors must be taken into account in model-based analyses of electrocatalytic activity.

The polarization curve is also affected by surface charging effects. The surface charging relation is nonmonotonic in the adsorption region (Figure S12) since we have considered the partially charged adsorbates (eq 22) in this model.^58^ Therefore, the increase of negative surface charge induced by the dipole moment of the adsorbates suppresses the activity by inhibiting the adsorption of formate (eqs 32 and 33). As shown in Figure S13, the simulated *j*–*E*_M_ curve transforms from a bell shape to a camel shape, with a valley in the adsorption region of HCOO_b_. This phenomenon is observed in the experiments of FAOR on a polycrystalline Pt electrode.^49^ However, it is not observed in experiments on a Pt(111) electrode.^43^ The reason for this discrepancy might be the oversimplification of the surface charging effect as accounted for in the Gibbs free energies of adsorbates. Detailed atomic calculations will be needed to describe this relation more accurately.

**Figure 10:**
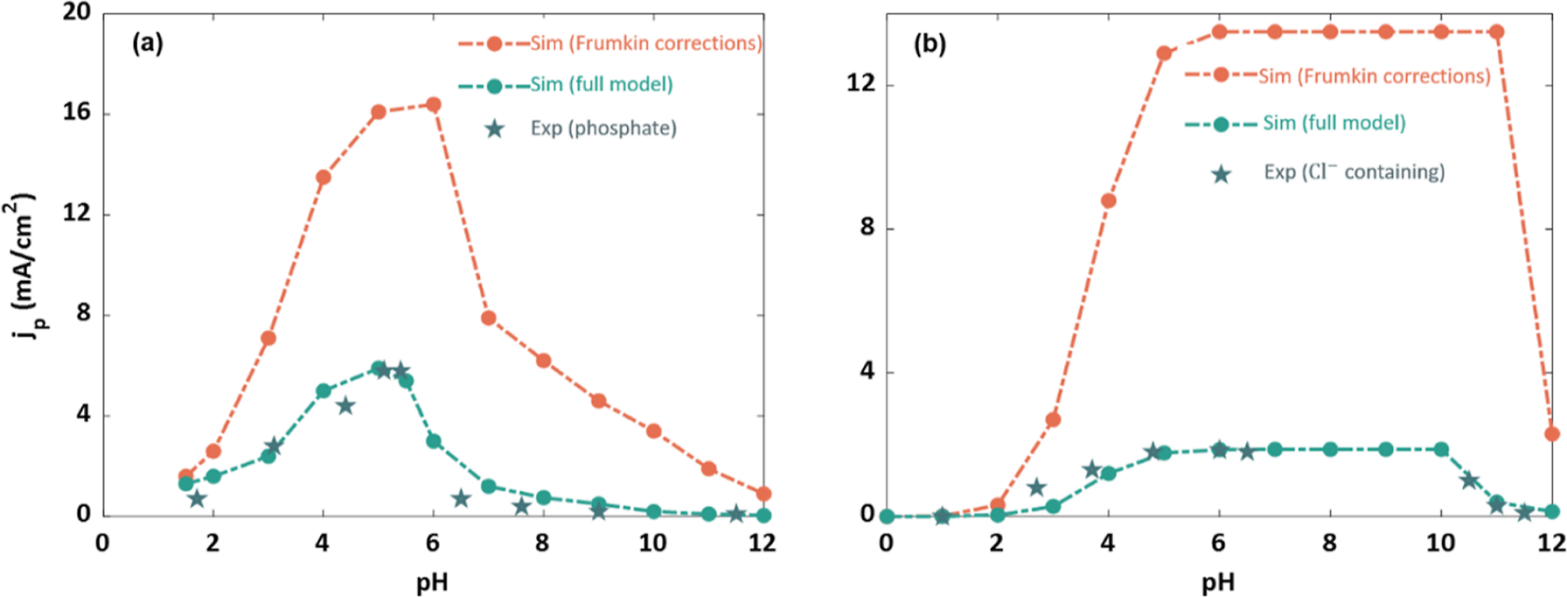
Comparison of the simulated *j*_p_–pH relations and the experimental *j*_p_–pH relations in (a) 0.2 M phosphate solution and (b) 0.5 M perchlorate + 10 mM chloride solution. The temperature is 20 °C in phosphate solution and 25 °C in chloride-containing solution in experiments. The concentration of formic acid is 0.05 M in phosphate solution and 0.1 M in chloride-containing solution. Experimental data for Pt(111) in phosphate solution are taken from ref (51) and those for polycrystalline Pt in chloride-containing solution from ref (45).

We have furthermore examined our model by comparison with experimental data for formic acid oxidation in phosphate and chloride-containing solutions. Simulations done with the full model closely reproduce the experimental data, as can be seen in Figure 10. The quantitative agreement demonstrates that the full model reliably captures the interplay of intrinsic kinetics, mass transport, and surface charging effects in different solutions.

## Discussion

In this section, we first provide a critical account of various explanations for the peculiar pH effects in the FAOR. Then, we address limitations of the model and discuss extensions needed to generalize the model.

### Critical Review of Existing Explanations

There have been several explanations for the pH effects in FAOR. Joo *et al.* observed a bell-shaped *j*_p_–pH relation on Pt with the peak at p*K*_a_. They explained this phenomenon as follows. When pH < p*K*_a_, the concentration of HCOO^–^ increases with pH, the activity increases since HCOO^–^ is the main reactant. When pH > p*K*_a_, the site-blocking effect of OH_ad_ adsorption decreases the activity.^24^ Brimaud *et al.* also obtained a bell-shaped relation on Au. However, the OH_ad_ adsorption is negligible on Au in the potential range of interest. They then proposed that the main reactant is not HCOO^–^, but the [HCOOH–HCOO^–^] dimer, which has the highest concentration at p*K*_a_.^49^

However, recent experiments have shown that the *j*_p_–pH relation in perchlorate/sulfate solutions presents a trapezoidal shape with a plateau between pH 5 and 10.^26,45^ Neither of the above two mechanisms can explain this phenomenon. Perales-Rondón *et al.* noticed that the bell-shaped *j*_p_–pH relation in ref (24) is obtained in phosphate solution. Therefore, they stressed the site-blocking effect of adsorbed phosphate anions and attributed the plateau region to the constant concentration of reactant HCOO^–^.^45,49^ In contrast, Zhang *et al.* proposed that the *j*_p_–pH relation in phosphate solution is intrinsic due to the buffering capacity of the electrolyte, while the site-blocking effect of adsorbed phosphate anions is not important. They conjectured that the plateau region in non-buffered perchlorate solution might be caused by the local pH shift.^25^ However, because their model did not include the effects of local pH and reactant concentration on the microkinetics, they were unable to find a consistent physical explanation of the experimental observation.

In addition, the activity, which is proportional to , is not solely determined by the reactant concentration or the surface pH. There are three factors, the rate constant, the reactant concentration, and the abundance of free surface sites, that control the activity. Specifically, the rate constant, , wherein the driving force is defined as *E*_M,RHE_ – 0.059pH_bulk_ – φ_RP_, does not explicitly depend on the surface pH but the bulk pH of the solution. In addition, φ_RP_, which is controlled by the surface charge density, changes with pH (Figure S14). The reactant concentration is not only dictated by the surface pH since the dissociation reaction of a weak acid does not remain in equilibrium during the reaction process.^70^ As shown in Figure S15, the water dissociation deviates from the equilibrium state when pH = 12. The coverage of free sites  depends not only on OH_ad_ adsorption but also on HCOO_b_ adsorption. By combining microkinetic modeling and LRE effects, we resolve the discrepancy on the pH effects in FAOR: (1) the intrinsic kinetic *j*_p_–pH relation exhibits a bell shape with the peak at a pH close to 6; (2) in perchlorate solution, the surface pH remains constant around 4 between pH 5 and 10 due to the mass transport, which inhibits the adsorption of OH_ad_ and hereby leads to constant , *E*_p,SHE_ and θ_0_, thus resulting in the plateau region; (3) in phosphate solution, the site-blocking effect of adsorbed phosphate is negligible when pH < 4 and becomes significant when pH > 6, resulting in a bell-shaped *j*_p_–pH relation with the peak at pH = 6, but this is not the intrinsic kinetic *j*_p_–pH relation; in perchlorate solution containing Cl^–^, the site-blocking effect of Cl^–^ is significant at any pH, resulting in a plateau region between pH 6 and 10; and (4) the increasing negative surface charge density with pH suppresses the activity. The latter three aspects further support the crucial message that proper consideration of the LRE is important to correctly predict pH effects of electrocatalytic reactions.

From the model presented in this work, suggestions for improving the activity of the considered reaction can be derived: the electrolyte should be kept at pH = 6, the value that results in the highest intrinsic activity of the reaction; mass transport of formate should be enhanced because formate is the main reactant; buffer electrolytes with anion chemisorption, such as phosphate, should be avoided since chemisorbed anions block the active sites on the catalyst surface; and the potential of zero charge of the catalyst should be shifted to more negative value because a negative surface charge density inhibits the activity.

### Limitations and Extensions

This section aims at putting this work into a wider context and underpinning its general significance. pH effects are ubiquitous in electrocatalysis, and they play various roles, broadly categorized into intrinsic pH effects and pH-dependent LRE. Intrinsic pH effects include the shifts in potential and reactant concentration with pH. These two factors were incorporated into a kinetic model to reproduce the volcano-shaped activity–pH relation on the RHE scale.^19^ The more subtle LRE effects have been widely neglected until recently. Zhang *et al.*(25) and Lamoureux *et al.*(14) stressed the importance of mass transport on understanding pH effects. Compared to mass-transport phenomena, surface charging phenomena are more complex. The classical approach of handling surface charging effects is *via* Frumkin corrections, which describe the potential and concentration distributions in the local reaction zone. With the development of advanced experimental and theoretical tools, other surface charging effects beyond Frumkin corrections have been proposed recently. Lamoureux *et al.*,^14^ Liu *et al.*,^32^ and Kelly *et al.*(30) emphasized the impact of the electric field on adsorption energies of intermediates. The impact of surface charging on interfacial solvent reorganization energies has also been highlighted.^33,34,71^ In addition, impacts of surface charging on activation barriers and position of the RP were proposed recently.^72^

**Figure 11:**
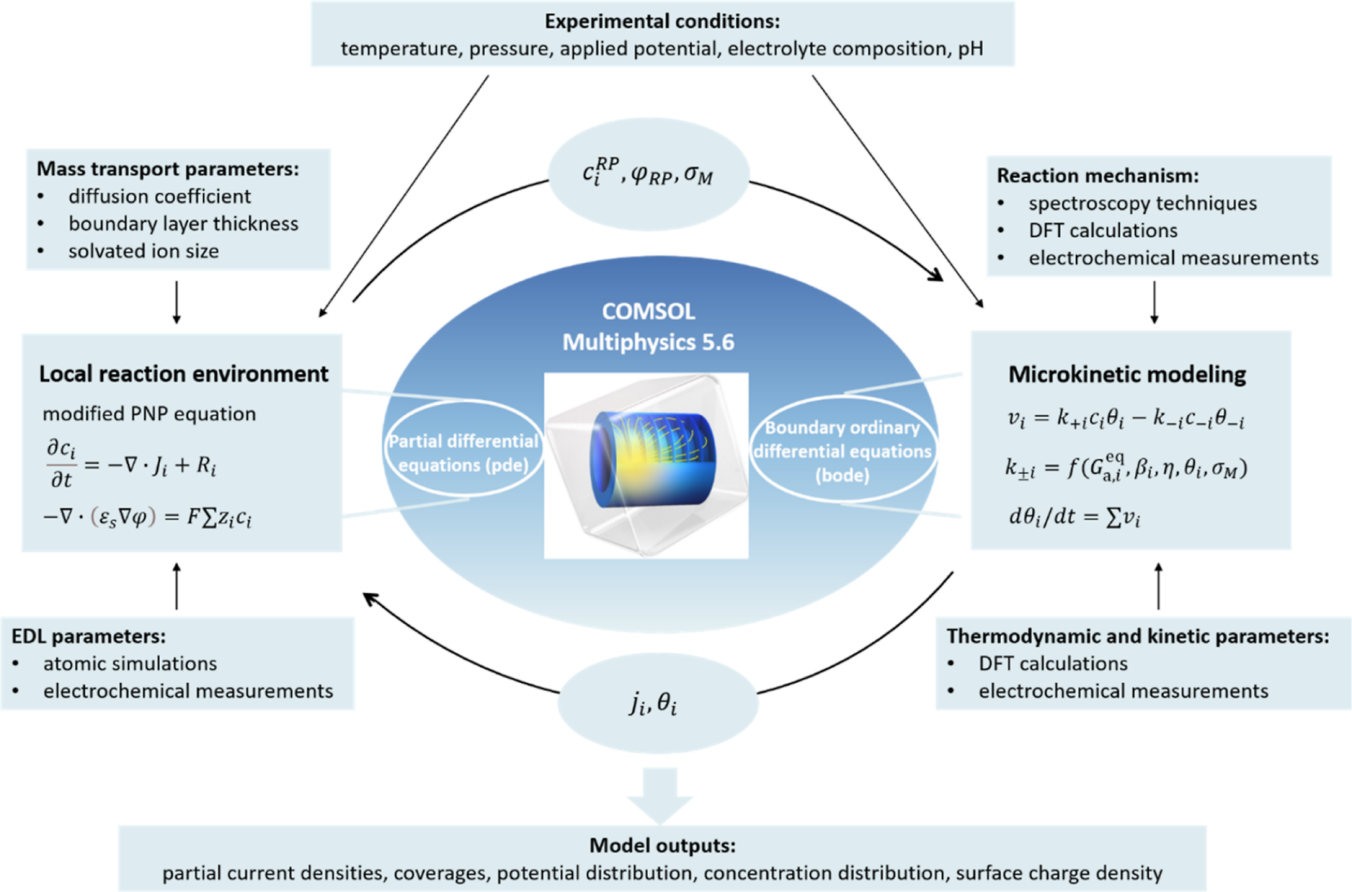
Flowchart for studying common electrochemical reactions with the framework presented in this work.

A change in the solution pH affects the reaction conditions and kinetics in many ways. The framework used in this work can disentangle these coupled effects. The presented framework is general, and it can be applied to decipher pH effects in other electrocatalytic reactions. To do so, the first step should be to determine the reaction mechanism of the reaction of interest and based that on build the microkinetic model with the support of DFT calculations. Analytic treatment in this step is helpful to understand the reaction metrics. Efforts along these lines have been made to understand the rate-determining term^73^ and the Tafel slope of various electrocatalytic reactions.^74^ The second step is to set up the model system and then build the LRE model. The mass-transport parameters, such as the diffusion layer thickness and diffusion coefficients, must be determined corresponding to the experimental conditions. Furthermore, EDL parameters, such as the configuration of adlayer and the net charge number of adsorbates, are needed, which can be obtained with the help of atomistic calculations. The flowchart for studying common electrochemical reactions using this framework is presented in Figure 11.

However, in the framework presented in this article, several assumptions and approximations have been made. First, we have used the Butler–Volmer description for the electron-transfer kinetics. However, the rate constant increases infinitely with overpotential in the Butler–Volmer approach, which is unrealistic at high overpotentials.^75^ Advanced electron-transfer theories, for example, the Marcus–Hush theory, can be used to upgrade this framework.^75^ In addition, Marcus–Hush theory allows for the inclusion of interfacial solvent reorganization energy. Second, we have treated the EDL as a serial connection of an adlayer and a diffuse layer. Atomic-scale understanding from first-principles calculations can be used to obtain parameters of the EDL model.^76,77^ Third, we have assumed the surface charging effect on the adsorption energy of intermediate simply as a linear relation. DFT-based calculations should be employed to gain explicit insights into this relation.^57,68^ Fourth, the steady-state assumption has been used in this work. In future work, dynamic pH effects should be accounted for.

## Conclusions

In summary, we have presented a hierarchical framework to understand the pH effects in electrocatalytic reactions. Capabilities of this framework have been examined by studying the pH effects of FAOR. We have shown that the intrinsic activity–pH relation of FAOR exhibits a bell shape, but the peak is located at pH = 6, rather than at the p*K*_a_ of formic acid, deviating from previous wisdom. Mass-transport effects, which keep the surface pH close to 4 for 5 < pH < 10 and thus inhibit OH_ad_ adsorption, induce the activity–pH relation to transform from a bell shape to a trapezoidal shape. We have attributed the bell-shaped activity–pH relation with the peak at pH = 6 in phosphate solution to the strong adsorption of phosphate anions. The Frumkin corrections include two competing factors, *i.e.*, increasing (decreasing) the driving force and decreasing (increasing) the reactant concentration at the surface. These two factors more or less cancel each other off in this case and thus do not markedly affect the activity–pH relations. By considering the surface charging effects on the free energies of intermediates, we have obtained quantitative agreement with experimental finding. Though this work focuses on the FAOR, the framework used in this work is general and readily applicable to other electrocatalytic reactions, as has been shown.

## Supporting Information Available

Details of microkinetic modeling; LRE modeling; model parameters; effect of lateral interactions on the analytical solution; adsorption isotherm of HCOO_b_; buffering effects of phosphate solution; sensitivity of diffusion layer thickness; surface charging relations; and additional simulation data (PDF)
