# Supplementary material for: pH Effects in a Model Electrocatalytic Reaction Disentangled
Source: JACS Au. 2023 Mar 1;3(4):1052–64. doi: 10.1021/jacsau.2c00662 (PMC10131201; doi:10.1021/jacsau.2c00662)
Supplement: Supplementary file 1 — au2c00662_si_001.pdf [file au2c00662_si_001.pdf]

Supporting Information

pH effects in a model electrocatalytic reaction disentangled

Xinwei Zhu,[a,b] Jun Huang,[a] Michael Eikerling,[a,b,*]

[a] *Theory and Computation of Energy Materials (IEK-13), Institute of Energy and Climate Research, Forschungszentrum Jülich GmbH, 52425 Jülich, Germany*

[b] *Chair of Theory and Computation of Energy Materials, Faculty of Georesources and Materials Engineering, RWTH Aachen University, 52062 Aachen, Germany*

** corresponding author, email:*

Michael Eikerling: m.eikerling@fz-juelich.de

ORCID:

Xinwei Zhu: 0000-0002-4636-8893

Jun Huang: 0000-0002-1668-5361

Michael Eikerling: 0000-0002-0764-8948

Supplementary Note 1: Details of the microkinetic modeling

For the reaction rates, we have the following relations,

$$v_1 = k_1 c^{\mathrm{RP}}_{\mathrm{HCOOH}} \theta_0^2 - k_{-1} \theta_{\mathrm{HCOO_b}} c^{\mathrm{RP}}_{\mathrm{H^+}} \tag{S1}$$

$$v_2 = k_2 c^{\mathrm{RP}}_{\mathrm{HCOO^-}} \theta_0^2 - k_{-2} \theta_{\mathrm{HCOO_b}} \tag{S2}$$

$$v_3 = k_3 c^{\mathrm{RP}}_{\mathrm{HCOOH}} \theta_0 - k_{-3} \theta_{\mathrm{HCOO_m}} c^{\mathrm{RP}}_{\mathrm{H^+}} \tag{S3}$$

$$v_4 = k_4 c^{\mathrm{RP}}_{\mathrm{HCOO^-}} \theta_0 - k_{-4} \theta_{\mathrm{HCOO_m}} \tag{S4}$$

$$v_5 = k_5 \theta_{\mathrm{HCOO_m}} \tag{S5}$$

$$v_6 = k_6 \theta_0 - k_{-6} \theta_{\mathrm{OH_{ad}}} c^{\mathrm{RP}}_{\mathrm{H^+}} \tag{S6}$$

$$v_7 = k_7 c^{\mathrm{RP}}_{\mathrm{OH^-}} - k_{-7} \theta_{\mathrm{OH_{ad}}} \tag{S7}$$

$$v_8 = k_8 c^{\mathrm{RP}}_{\mathrm{HPO_4^{2-}}} - k_{-8} \theta_{\mathrm{HPO_4^-}{}_{\mathrm{ad}}} \tag{S8}$$

$$v_9 = k_9 c^{\mathrm{RP}}_{\mathrm{Cl^-}} - k_{-9} \theta_{\mathrm{Cl_{ad}}} \tag{S9}$$

where $\theta_0 = \theta_{\mathrm{max}} - 2\theta_{\mathrm{HCOO_b}} - \theta_{\mathrm{HCOO_m}} - \theta_{\mathrm{OH_{ad}}} - \theta_{\mathrm{HPO_4^-}{}_{\mathrm{ad}}} - \theta_{\mathrm{Cl_{ad}}}$ is the coverage of vacancy (free sites), with $\theta_{\mathrm{max}} = 0.6$ being the maximum $2\theta_{\mathrm{HCOO_b}}$;[1] $c^{\mathrm{RP}}_{\mathrm{HCOOH}}$, $c^{\mathrm{RP}}_{\mathrm{H^+}}$, $c^{\mathrm{RP}}_{\mathrm{HCOO^-}}$, $c^{\mathrm{RP}}_{\mathrm{OH^-}}$, $c^{\mathrm{RP}}_{\mathrm{HPO_4^{2-}}}$ and $c^{\mathrm{RP}}_{\mathrm{Cl^-}}$ are concentrations at the reaction plane that need to be determined from the PNP model. It should be noted that we use the same θ_{max} for all adsorbates, while the θ_{max} for $\mathrm{OH_{ad}}$ in ref. [2] is about 0.3. Therefore, we examine the sensitivity of $\theta_{\mathrm{OH}}^{\mathrm{max}}$ in the $j - \mathrm{E_M}$ curve at pH values of 3, 7 and 11, corresponding to acidic, neutral and alkaline conditions, respectively. The results in Figure S1 show that the $j - \mathrm{E_M}$ curve is not sensitive to $\theta_{\mathrm{OH}}^{\mathrm{max}}$ at acidic pH, while the decrease in $\theta_{\mathrm{OH}}^{\mathrm{max}}$ weakens the site-blocking effect of $\mathrm{OH_{ad}}$ at neutral/alkaline pH and high potentials.

However, the trend of the $j - \mathrm{E_M}$ curve remains same. Therefore, the simplification of using the same $\theta_{\max}$ for $\mathrm{HCOO_b}$ and $\mathrm{OH_{ad}}$ is reasonable for the case in this work.

We consider the variations in the Gibbs free energies of $\mathrm{HCOO_b}$, $\mathrm{HCOO_m}$, $\mathrm{OH_{ad}}$ and $\mathrm{HPO_4^-}_{\mathrm{ad}}$ due to lateral interactions between adsorbates,[3]

$$\Delta G_{\mathrm{HCOO_b}} = \xi_{\mathrm{HCOO_b-HCOO_b}} \theta_{\mathrm{HCOO_b}} \tag{S10}$$

$$\Delta G_{\mathrm{HCOO_m}} = \xi_{\mathrm{HCOO_b-HCOO_m}} \theta_{\mathrm{HCOO_b}} + \xi_{\mathrm{OH_{ad}-HCOO_m}} \theta_{\mathrm{OH_{ad}}} + \xi_{\mathrm{HPO_4^-}_{\mathrm{ad}}-\mathrm{HCOO_m}} \theta_{\mathrm{HPO_4^-}_{\mathrm{ad}}} \tag{S11}$$

$$\Delta G_{\mathrm{OH_{ad}}} = \xi_{\mathrm{OH_{ad}-OH_{ad}}} \theta_{\mathrm{OH_{ad}}} \tag{S12}$$

$$\Delta G_{\mathrm{HPO_4^-}_{\mathrm{ad}}} = \xi_{\mathrm{HPO_4^-}_{\mathrm{ad}}-\mathrm{HPO_4^-}_{\mathrm{ad}}} \theta_{\mathrm{HPO_4^-}_{\mathrm{ad}}} \tag{S13}$$

with ξ_{X-Y} the lateral interaction parameter. The variations in the reaction Gibbs free energy of steps are given by,

$$\Delta\Delta G_1 = \Delta\Delta G_2 = \Delta G_{\mathrm{HCOO_b}} \tag{S14}$$

$$\Delta\Delta G_3 = \Delta\Delta G_4 = -\Delta\Delta G_5 = \Delta G_{\mathrm{HCOO_m}} \tag{S15}$$

$$\Delta\Delta G_6 = \Delta\Delta G_7 = \Delta G_{\mathrm{OH_{ad}}} \tag{S16}$$

$$\Delta\Delta G_8 = \Delta G_{\mathrm{HPO_4^-}_{\mathrm{ad}}} \tag{S17}$$

All the parameters included in the microkinetic model are listed in Supplementary Tables 1 and 2.

According to the thermodynamic equilibrium, we have

$$E_1^{\mathrm{eq}} = E_2^{\mathrm{eq}} + \frac{\Delta \mathrm{G}^0_{\mathrm{HCOO^-}} + \Delta \mathrm{G}^0_{\mathrm{H^+}} - \Delta \mathrm{G}^0_{\mathrm{HCOOH}}}{\mathrm{e}} \tag{S18}$$

According to the dissociation equilibrium of formic acid, $\mathrm{HCOOH} \rightleftarrows \mathrm{HCOO^-} + \mathrm{H^+}$ ($\mathrm{pK_a} = 3.75$),

we have

$$\Delta G^0_{\mathrm{HCOOH}} = \Delta G^0_{\mathrm{HCOO^-}} + \Delta G^0_{\mathrm{H^+}} + \mathrm{RTln}(10^{-\mathrm{pK_a}}) \tag{S19}$$

Combining Eqs. S18 and S19, the relations between the standard equilibrium potentials of Steps 1 and 2 can be determined,

$$E_1^{\mathrm{eq}} = E_2^{\mathrm{eq}} + 0.22\ \mathrm{V} \tag{S20}$$

Following the same logic, we obtain

$$E_3^{\mathrm{eq}} = E_4^{\mathrm{eq}} + 0.22\ \mathrm{V} \tag{S21}$$

$$E_5^{\mathrm{eq}} = -E_3^{\mathrm{eq}} - 0.07\ \mathrm{V} \tag{S22}$$

$$E_6^{\mathrm{eq}} = E_7^{\mathrm{eq}} + 0.83\ \mathrm{V} \tag{S23}$$

The $\mathrm{HCOO_b}$ adsorption is very fast and in equilibrium, as shown in voltammogram.[4] Therefore, the activation barriers of $\mathrm{HCOO_b}$ adsorption steps should be very low. We give $G^{\mathrm{eq}}_{\mathrm{a,1}} = G^{\mathrm{eq}}_{\mathrm{a,2}}$ $= 0.3$ eV, close to the DFT calculations.[5] $E_1^{\mathrm{eq}} = 0.4\ \mathrm{V}$ is taken from DFT results,[5,6] which is consistent with the peak position of $\mathrm{HCOO_b}$ adsorption.[4] $E_2^{\mathrm{eq}} = 0.18\ \mathrm{V}$ is calculated based on Eq. S20. $\beta_1 = \beta_2 = 0.5$ is taken from DFT study.[5] Using transient calorimetry, $\Delta G^0_{\mathrm{HCOO_m}} - \Delta G^0_{\mathrm{HCOO_b}}$ $= 0.3$ eV is obtained.[7] Therefore, $E_3^{\mathrm{eq}} = E_1^{\mathrm{eq}} + 0.3 = 0.7\ \mathrm{V}$, $E_4^{\mathrm{eq}} = E_3^{\mathrm{eq}} - 0.22 = 0.48\ \mathrm{V}$, $E_5^{\mathrm{eq}} = -E_1^{\mathrm{eq}} - 0.07 = -0.77\ \mathrm{V}$. The oxidation of $\mathrm{HCOO_m}$ is much faster than its adsorption due to its negligible coverage, implying that the adsorption Steps 3 and 4 are rate determining steps. Therefore, $\beta_3 = \beta_4$ is set to be 0.4, corresponding to the value estimated from Tafel slope (150 mV/dec).[8] The sensitivity of β_4 in the polarization curve at pH = 1 is shown in Figure S3. $G^{\mathrm{eq}}_{\mathrm{a,3}}$ and $G^{\mathrm{eq}}_{\mathrm{a,4}}$ are fitted in the experimentally determined range of apparent activation barrier (0.4 – 0.6 eV).[9] $G^{\mathrm{eq}}_{\mathrm{a,5}}$ and β_5 are not sensitive parameters. β_5 is set to usual value 0.5, $G^{\mathrm{eq}}_{\mathrm{a,5}}$ is given as 0.6 eV

(the activation barrier is low in the interested potential range since E_5^{eq} is very negative). For the $\mathrm{OH_{ad}}$ adsorption, we adopt DFT calculated results.[10,11] For the lateral interaction parameters, $\xi_{\mathrm{HCOO_b-HCOO_b}}$ was calculated to be 0.2 eV,[1] $\xi_{\mathrm{OH_{ad}-OH_{ad}}}$ is set to be 0.15 eV,[12] $\xi_{\mathrm{HCOO_b-HCOO_m}}$ and $\xi_{\mathrm{OH_{ad}-HCOO_m}}$ are fitted parameters. For $\mathrm{HPO_4^{2-}}$ and $\mathrm{Cl^-}$ adsorption, we use $G_{\mathrm{a},8}^{\mathrm{eq}} = G_{\mathrm{a},9}^{\mathrm{eq}} = 0.3\ \mathrm{eV}$, implying the strong adsorption of these two anions. E_8^{eq} and E_9^{eq} correspond to the potentials at which the specific adsorption occurs.[13,14] $\beta_8 = \beta_9 = 0.5$ are usual values.

Supplementary Table 1. Thermodynamic and kinetic parameters used in the microkinetic model, including activation barrier $G_{a,i}^{eq}$, equilibrium potential E_i^{eq}, and transfer coefficient β_i

Step i	$G_{a,i}^{eq}$ (eV)	E_i^{eq} (V vs. SHE)	β_i
1	0.3	0.4	0.5
2	0.3	0.18	0.5
3	0.56	0.7	0.4
4	0.44	0.48	0.4
5	0.6	-0.77	0.5
6	0.26	0.78	0.5
7	0.26	-0.05	0.5
8	0.3	0.06	0.5
9	0.3	-0.04	0.5

Supplementary Table 2. Lateral interaction parameters (eV)

$\xi_{\mathrm{HCOO_b - HCOO_m}}$	0.2	$\xi_{\mathrm{HPO_4^-{}_{ad} - HCOO_m}}$	0.15
$\xi_{\mathrm{HCOO_b - HCOO_b}}$	0.2	$\xi_{\mathrm{HPO_4^-{}_{ad} - HPO_4^-{}_{ad}}}$	0.3
$\xi_{\mathrm{OH_{ad} - HCOO_m}}$	0.15	$\xi_{\mathrm{Cl_{ad} - HCOO_m}}$	0.15
$\xi_{\mathrm{OH_{ad} - OH_{ad}}}$	0.15	$\xi_{\mathrm{Cl_{ad} - Cl_{ad}}}$	0.25

Supplementary Note 2: Details of the local reaction environment modeling

Two homogeneous reactions, formic acid dissociation and water dissociation, are considered to occur in the perchlorate electrolyte

$$\mathrm{HCOOH} \rightleftharpoons \mathrm{H}^+ + \mathrm{HCOO}^- \ (\mathrm{pK_a} = 3.75) \quad \text{(Step S1)}$$

$$\mathrm{H_2O} \rightleftharpoons \mathrm{OH}^- + \mathrm{H}^+ \ (\mathrm{pK_a} = 14) \quad \text{(Step S2)}$$

The production rate of species i due to the homogeneous reactions, R_i, can be written as

$$R_{\mathrm{HCOOH}} = -k_{\mathrm{s1}} c_{\mathrm{HCOOH}} + k_{-\mathrm{s1}} c_{\mathrm{H^+}} c_{\mathrm{HCOO^-}} \quad \text{(S24)}$$

$$R_{\mathrm{HCOO^-}} = k_{\mathrm{s1}} c_{\mathrm{HCOOH}} - k_{-\mathrm{s1}} c_{\mathrm{H^+}} c_{\mathrm{HCOO^-}} \quad \text{(S25)}$$

$$R_{\mathrm{H^+}} = k_{\mathrm{s1}} c_{\mathrm{HCOOH}} - k_{-\mathrm{s1}} c_{\mathrm{H^+}} c_{\mathrm{HCOO^-}} + k_{\mathrm{s2}} - k_{-\mathrm{s2}} c_{\mathrm{H^+}} c_{\mathrm{OH^-}} \quad \text{(S26)}$$

$$R_{\mathrm{OH^-}} = k_{\mathrm{s2}} - k_{-\mathrm{s2}} c_{\mathrm{H^+}} c_{\mathrm{OH^-}} \quad \text{(S27)}$$

where k_{si} and $k_{-\mathrm{si}}$ are the rate constants of forward reaction and reverse reaction of step Si, respectively.

When we study the FAOR in phosphate solution, in addition to Steps S1 and S2, the following buffer reactions are considered

$$\mathrm{H_3PO_4} \rightleftharpoons \mathrm{H_2PO_4^-} + \mathrm{H}^+ \ (\mathrm{pK_a} = 2.16) \quad \text{(Step S3)}$$

$$\mathrm{H_2PO_4^-} \rightleftharpoons \mathrm{HPO_4^{2-}} + \mathrm{H}^+ \ (\mathrm{pK_a} = 7.21) \quad \text{(Step S4)}$$

$$\mathrm{HPO_4^{2-}} \rightleftharpoons \mathrm{PO_4^{3-}} + \mathrm{H}^+ \ (\mathrm{pK_a} = 12.32) \quad \text{(Step S5)}$$

In this case, the production rates R_i, are written as

$$R_{\mathrm{HCOOH}} = -k_{\mathrm{s1}} c_{\mathrm{HCOOH}} + k_{-\mathrm{s1}} c_{\mathrm{H^+}} c_{\mathrm{HCOO^-}} \tag{S28}$$

$$R_{\mathrm{HCOO^-}} = k_{\mathrm{s1}} c_{\mathrm{HCOOH}} - k_{-\mathrm{s1}} c_{\mathrm{H^+}} c_{\mathrm{HCOO^-}} \tag{S29}$$

$$\begin{aligned} R_{\mathrm{H^+}} = {} & (k_{\mathrm{s1}} c_{\mathrm{HCOOH}} - k_{-\mathrm{s1}} c_{\mathrm{H^+}} c_{\mathrm{HCOO^-}}) + (k_{\mathrm{s2}} - k_{-\mathrm{s2}} c_{\mathrm{H^+}} c_{\mathrm{OH^-}}) + (k_{\mathrm{s3}} c_{\mathrm{H_3PO_4}} - k_{-\mathrm{s3}} c_{\mathrm{H^+}} c_{\mathrm{H_2PO_4^-}}) \\ & + (k_{\mathrm{s4}} c_{\mathrm{H_2PO_4^-}} - k_{-\mathrm{s4}} c_{\mathrm{H^+}} c_{\mathrm{HPO_4^{2-}}}) + (k_{\mathrm{s5}} c_{\mathrm{HPO_4^{2-}}} - k_{-\mathrm{s5}} c_{\mathrm{H^+}} c_{\mathrm{PO_4^{3-}}}) \end{aligned} \tag{S30}$$

$$R_{\mathrm{OH^-}} = k_{\mathrm{s2}} - k_{-\mathrm{s2}} c_{\mathrm{H^+}} c_{\mathrm{OH^-}} \tag{S31}$$

$$R_{\mathrm{H_3PO_4}} = -k_{\mathrm{s3}} c_{\mathrm{H_3PO_4}} + k_{-\mathrm{s3}} c_{\mathrm{H^+}} c_{\mathrm{H_2PO_4^-}} \tag{S32}$$

$$R_{\mathrm{H_2PO_4^-}} = k_{\mathrm{s3}} c_{\mathrm{H_3PO_4}} - k_{-\mathrm{s3}} c_{\mathrm{H^+}} c_{\mathrm{H_2PO_4^-}} - k_{\mathrm{s4}} c_{\mathrm{H_2PO_4^-}} + k_{-\mathrm{s4}} c_{\mathrm{H^+}} c_{\mathrm{HPO_4^{2-}}} \tag{S33}$$

$$R_{\mathrm{HPO_4^{2-}}} = k_{\mathrm{s4}} c_{\mathrm{H_2PO_4^-}} - k_{-\mathrm{s4}} c_{\mathrm{H^+}} c_{\mathrm{HPO_4^{2-}}} - k_{\mathrm{s5}} c_{\mathrm{HPO_4^{2-}}} + k_{-\mathrm{s5}} c_{\mathrm{H^+}} c_{\mathrm{PO_4^{3-}}} \tag{S34}$$

$$R_{\mathrm{PO_4^{3-}}} = k_{\mathrm{s5}} c_{\mathrm{HPO_4^{2-}}} - k_{-\mathrm{s5}} c_{\mathrm{H^+}} c_{\mathrm{PO_4^{3-}}} \tag{S35}$$

The forward rate constants of the homogeneous reactions are listed in Supplementary Table 3, including the references that they have been extracted from. The reverse rate constants can be calculated as

$$k_{-\mathrm{si}} = k_{\mathrm{si}} 10^{\mathrm{pK_a}} \tag{S36}$$

For the phosphate buffer reactions, we take large rate constants, $k_{\mathrm{s3}} = k_{\mathrm{s4}} = k_{\mathrm{s5}} = 1 \times 10^6$, following the assumption in ref. [15]. We only consider the steric effect of $\mathrm{Na^+}$ in this work since only $\mathrm{Na^+}$ and $\mathrm{ClO_4{}^-}$ have considerable concentrations and $a^3_{\mathrm{Na^+}} \approx 12$ $a^3_{\mathrm{ClO_4^-}}$. In addition, the electrode is negatively charged in most pHs and $\mathrm{Na^+}$ is the counterion in the electric double layer (EDL). The parameters of the EDL are listed in Supplementary Table 5.

Supplementary Table 3. Forward rate constants of homogeneous reactions

Reaction	S1[16]	S2[17]	S3[15]	S4[15]	S5[15]
k_{si}	4×10^{5}	2.4×10^{-5}	1×10^{6}	1×10^{6}	1×10^{6}

Supplementary Table 4. Parameters used to describe the mass transport, including diffusion coefficients D_i, effective solvated diameter a_i, kinematic viscosity of solution v, and the rotation speed of the RDE ω

Category	Item	Value	Source
D_i (m²/s)	$HCOOH$	1.41×10^{-9}	ref. [18]
	$HCOO^{-}$	1.454×10^{-9}	ref. [18]
	OH^{-}	5.27×10^{-9}	ref. [18]
	H^{+}	9.311×10^{-9}	ref. [18]
	Na^{+}	1.334×10^{-9}	ref. [19]
	ClO_4^{-}	1.029×10^{-9}	ref. [20]
	H_3PO_4	9.59×10^{-10}	ref. [21]
	$H_2PO_4^{-}$	8.5×10^{-10}	ref. [22]
	HPO_4^{2-}	6.9×10^{-10}	ref. [22]
	PO_4^{3-}	6.1×10^{-10}	ref. [22]
a_i (m)	Na^{+}	0.716×10^{-9}	ref. [19]
v (m²/s)	kinematic viscosity of water	1.1×10^{-6}	ref. [21]
ω (rpm)	rotation speed of the RDE	1000	ref. [23]

Supplementary Table 5. Parameters of the EDL

Category	Item	Value	Source
net charge number per adsorbate	$\varsigma_{\mathrm{HCOO_b}}$	0.03	fitted
	$\varsigma_{\mathrm{OH_{ad}}}$	0.04	fitted
	$\varsigma_{\mathrm{HPO_4^-}\ \mathrm{ad}}$	0.06	fitted
	$\varsigma_{\mathrm{Cl_{ad}}}$	0.02	fitted
permittivity of the vacuum	ε_0	8.85×10^{-12}	constant
permittivity of the solution	ε_s	$78.5\ \varepsilon_0$	permittivity of water
permittivity of the adlayer	ε_RP	$2.3\ \varepsilon_0$	$2-10\ \varepsilon_0$ [24]
thickness of the adlayer	δ_RP	$0.3\ \mathrm{nm}$	Pt-C distance of $\mathrm{HCOO_b}$ [25]
potential of zero charge	φ_pzc	0.3 V (SHE)	ref. [26]

Supplementary Note 3: Supporting simulation results

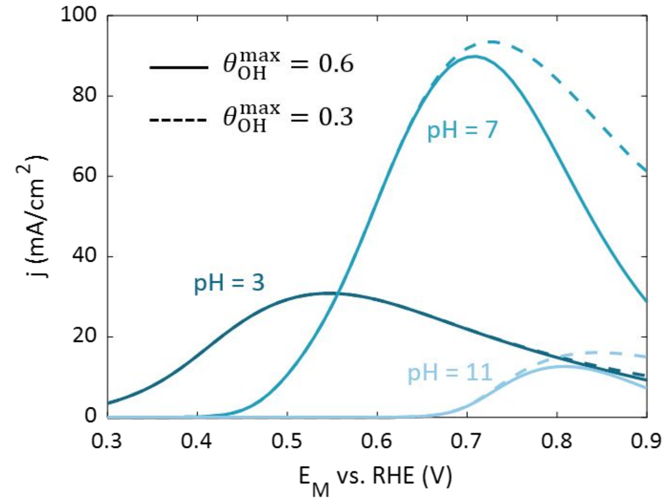

Figure S1. Sensitivity of $\theta_{\mathrm{OH}}^{\mathrm{max}}$ in the $j - E_{\mathrm{M}}$ curve at pH = 3, pH = 7 and pH = 11, corresponding to acidic, neutral and alkaline conditions, respectively.

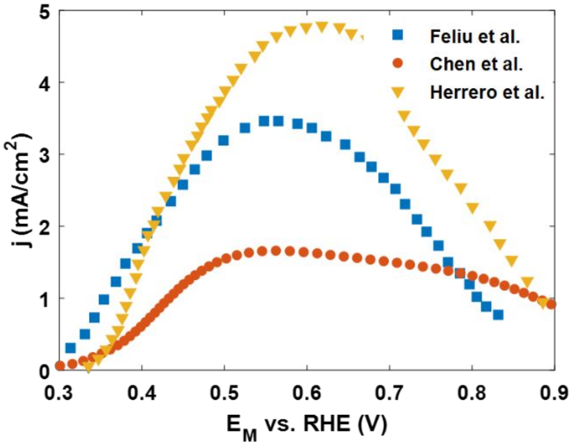

Figure S2. Experimental polarization curves of the oxidation of 0.1 M formic acid on Pt(111) from Feliu et al.,[9] Chen et al.,[27] and Herrero et al.[28] . The solution used in ref. [9] and ref. [28] is 0.1 M $HClO_4$, in ref. [27] is 0.1 M H_2SO_4.

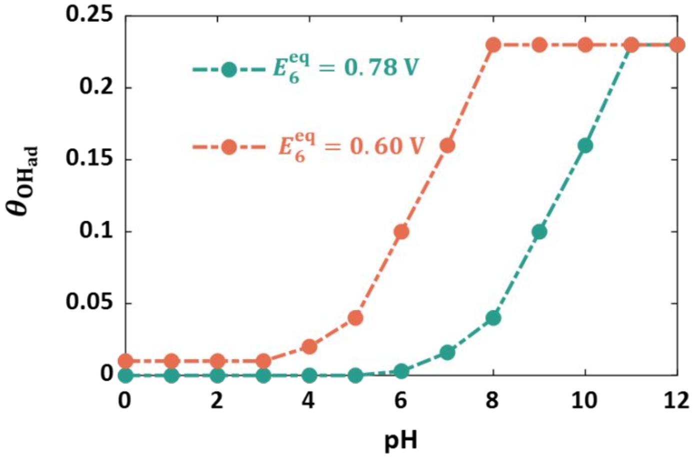

Figure S3. Coverage of OH_{ad} at peak potentials for $E_6^{eq} = 0.78$ V and $E_6^{eq} = 0.6$ V.

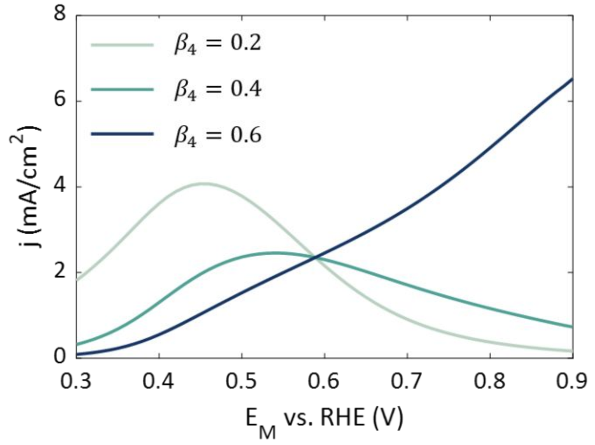

Figure S4. Sensitivity of β_4 in the $j - E_M$ curve at pH = 1. To assess the sensitivity, we vary the value of β_4 while keeping all other parameters constant, and then simulate the $j - E_M$ curves. The $j - E_M$ curve is bell-shaped only when $\beta_4 < 0.5$, as we predicted in Eq 30. In addition, the peak potential of the bell-shaped $j - E_M$ curve shifts toward a more negative potential as β_4 decreases.

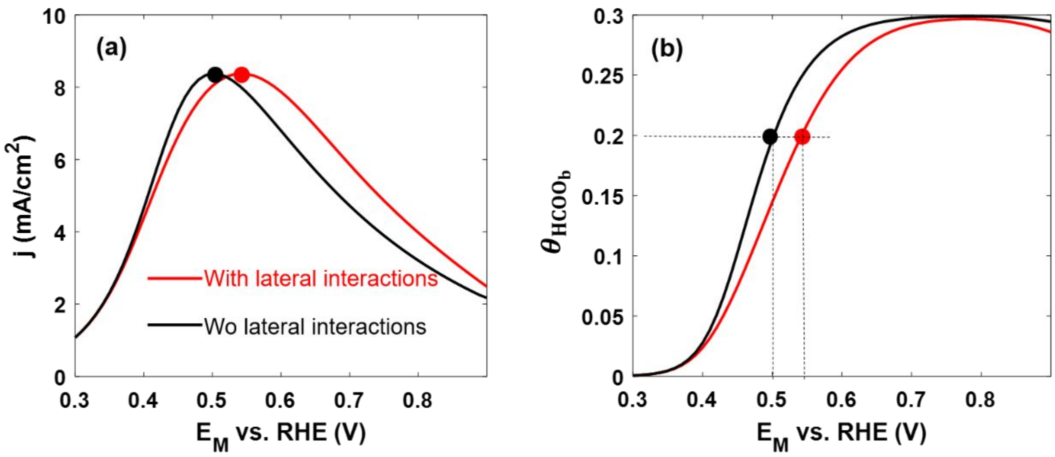

Figure S5. (a) Simulated $j-E_{\mathrm{M}}$ curves with and without considering lateral interactions at pH = 2. The peak potentials are indicated by dots. (b) Simulated coverages of $\mathrm{HCOO_b}$ with and without considering lateral interactions. In both cases, $\theta_{\mathrm{HCOO_b}}=\frac{1}{2}\frac{\beta_4}{1-\beta_4}\theta_{\max}=0.2$ is valid at the peak potentials.

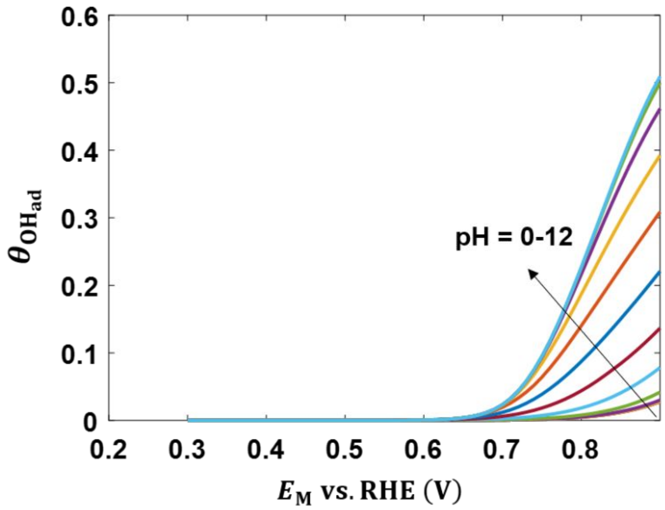

Figure S6. Adsorption isotherm of $\mathrm{OH_{ad}}$ in the pH range of 0 and 12 without considering the effects of mass transport.

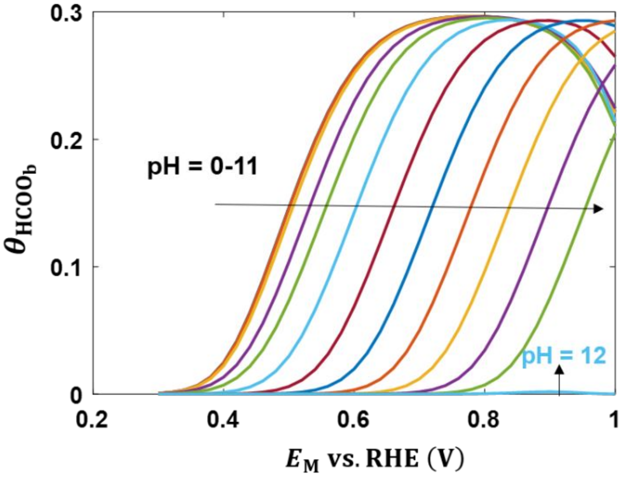

Figure S7. Adsorption isotherm of $HCOO_b$ in the pH range of 0 and 12 in the case of considering mass transport effects.

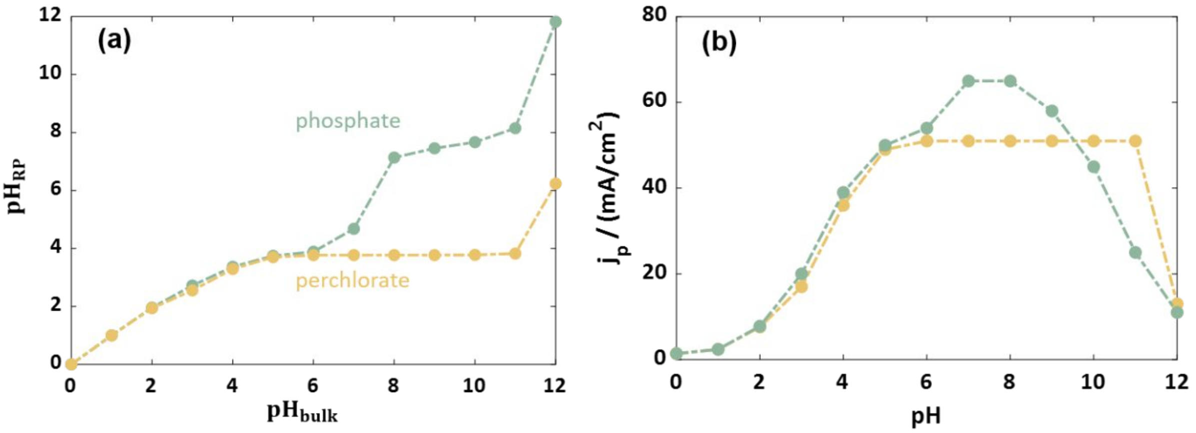

Figure S8. (a) Comparison of the surface pH at peak potentials and (b) comparison of the $j_p - pH$ relations in phosphate solutions and perchlorate solutions. The specific adsorption of phosphate anions is not considered in this case.

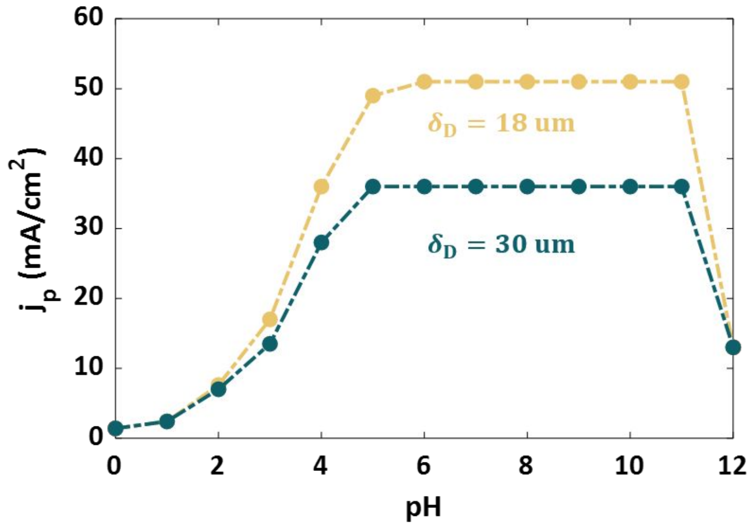

Figure S9. Sensitivity of the diffusion layer thickness δ_D in the mass transport corrected $j_\mathrm{p} - \mathrm{pH}$ relations.

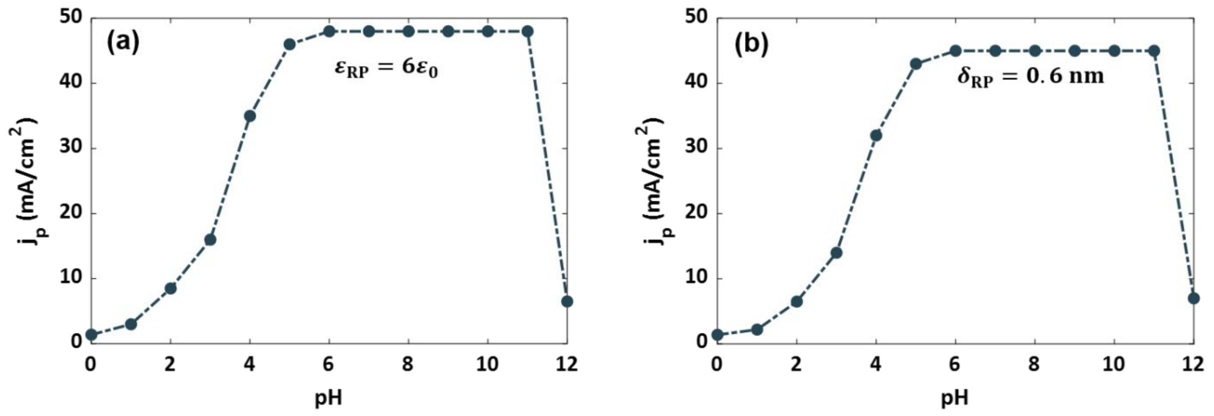

Figure S10. Simulated $j_\mathrm{p} - \mathrm{pH}$ relations when (a) $\varepsilon_\mathrm{RP} = 6\varepsilon_0$ and (b) $\delta_\mathrm{RP} = 0.6$ nm. Other parameters remain unchanged.

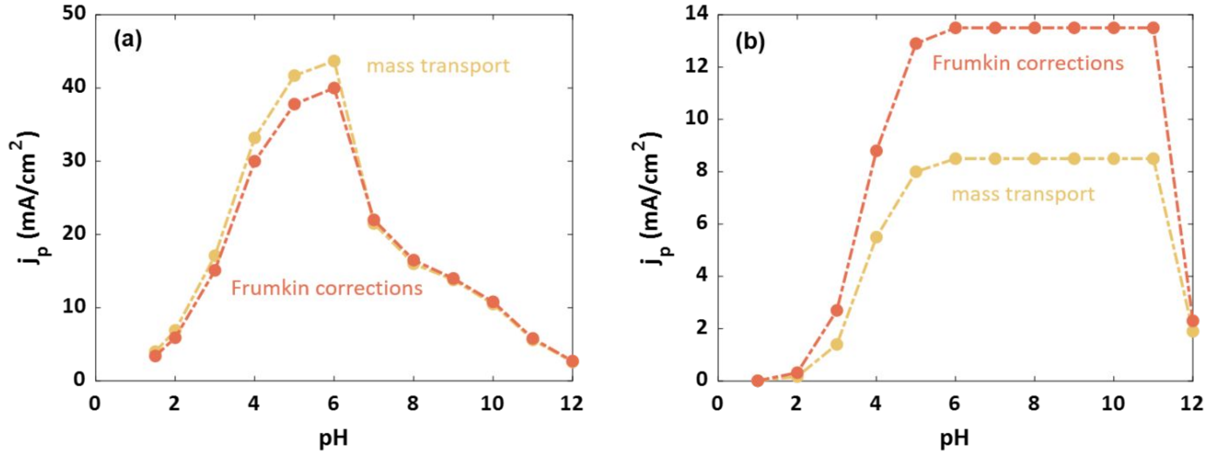

Figure S11. Comparison of the $j_{\mathrm{p}} - \mathrm{pH}$ relations corrected by only mass transport effects and the $j_{\mathrm{p}} - \mathrm{pH}$ relations corrected by Frumkin corrections in (a) phosphate solution and (b) chloride-containing solution.

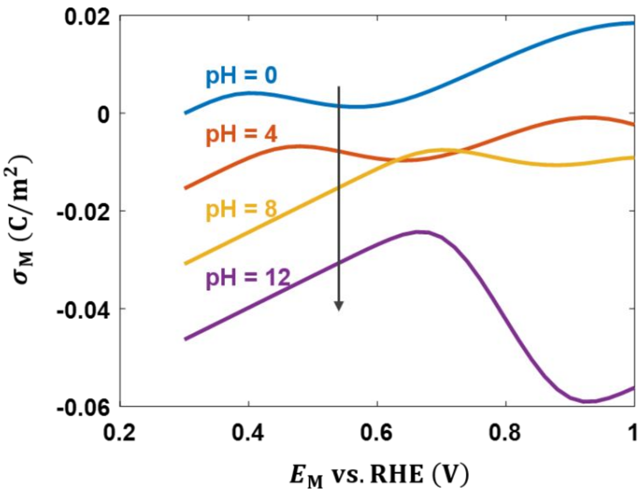

Figure S12. Surface charging relations at different pHs. The nonmonotonic features are caused by the chemisorption of $\mathrm{HCOO_b}$ and $\mathrm{OH_{ad}}$.[29]

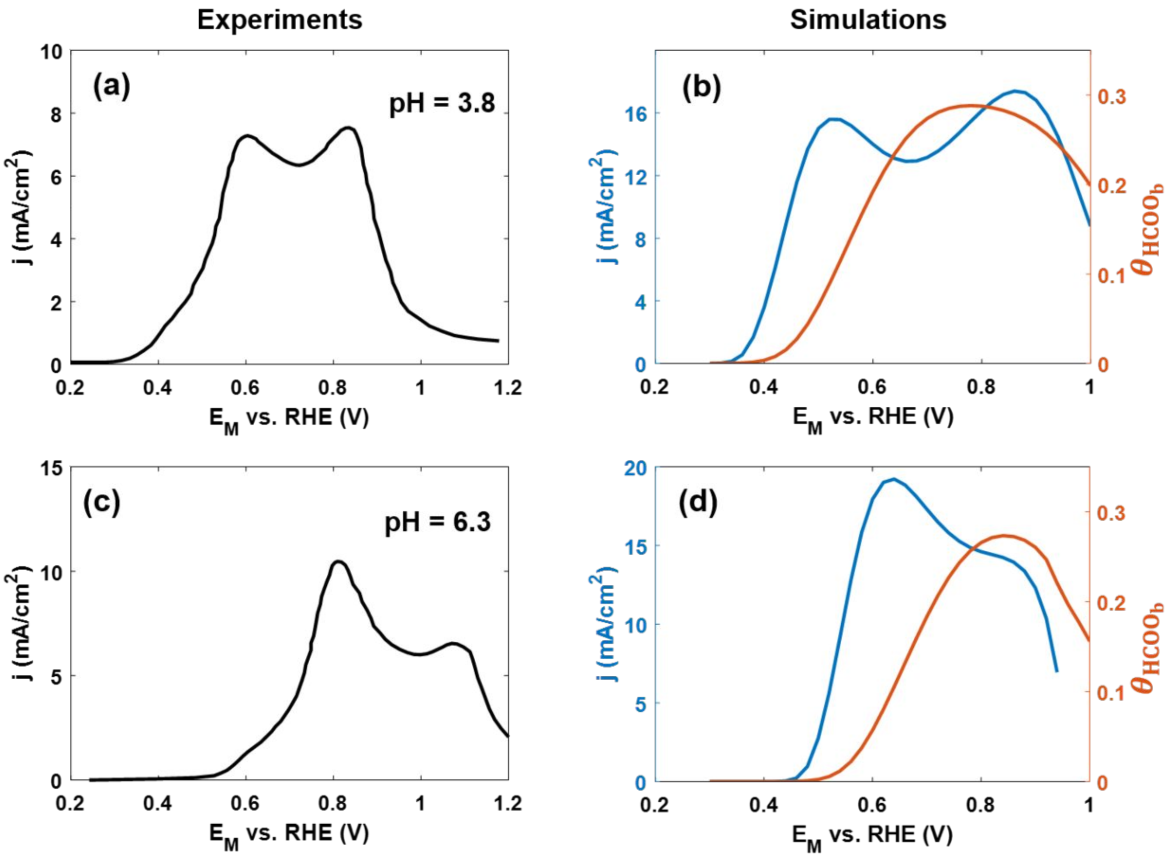

Figure S13. Comparisons of the experimental, (a) and (c), and the simulated, (b) and (d), $j - E_{\mathrm{M}}$ curves at pH = 3.8, (a) and (b), and pH = 6.3, (c) and (d). The simulated coverages of $\mathrm{HCOO_b}$ are also shown in (b) and (d). The experiments, corresponding to the negative scanning curves of potentiodynamic oxidation of 0.1 M HCOOH on a polycrystalline Pt bead electrode are taken from ref. [30].

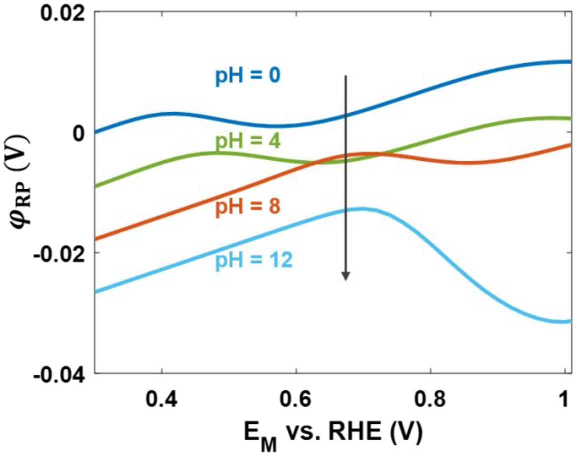

Figure S14. Potential at the reaction plane, φ_{RP}, at different E_M and pHs.

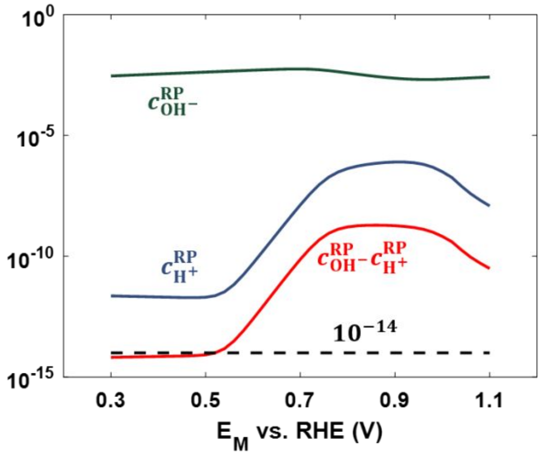

Figure S15. $c_{\mathrm{H}^+}^{\mathrm{RP}}$, $c_{\mathrm{OH}^-}^{\mathrm{RP}}$, and $c_{\mathrm{H}^+}^{\mathrm{RP}} c_{\mathrm{OH}^-}^{\mathrm{RP}}$ at pH = 12. $c_{\mathrm{H}^+} c_{\mathrm{OH}^-} = 10^{-14}$ if the water dissociation is in equilibrium, as indicated by the dashed line.

References

(1) Chen, X.; Granda-Marulanda, L. P.; McCrum, I. T.; Koper, M. T. M. How Palladium Inhibits CO Poisoning during Electrocatalytic Formic Acid Oxidation and Carbon Dioxide Reduction. *Nat. Commun.* **2022**, *13* (1), 38. https://doi.org/10.1038/s41467-021-27793-5.

(2) Wakisaka, M.; Udagawa, Y.; Suzuki, H.; Uchida, H.; Watanabe, M. Structural Effects on the Surface Oxidation Processes at Pt Single-Crystal Electrodes Studied by X-Ray Photoelectron Spectroscopy. *Energy Environ. Sci.* **2011**, *4* (5), 1662–1666. https://doi.org/10.1039/C0EE00756K.

(3) Liu, X.; Schlexer, P.; Xiao, J.; Ji, Y.; Wang, L.; Sandberg, R. B.; Tang, M.; Brown, K. S.; Peng, H.; Ringe, S.; Hahn, C.; Jaramillo, T. F.; Nørskov, J. K.; Chan, K. PH Effects on the Electrochemical Reduction of CO(2) towards C2 Products on Stepped Copper. *Nat. Commun.* **2019**, *10* (1), 32. https://doi.org/10.1038/s41467-018-07970-9.

(4) Grozovski, V.; Vidal-Iglesias, F. J.; Herrero, E.; Feliu, J. M. Adsorption of Formate and Its Role as Intermediate in Formic Acid Oxidation on Platinum Electrodes. *ChemPhysChem* **2011**, *12* (9), 1641–1644. https://doi.org/10.1002/cphc.201100257.

(5) Sakong, S.; Groß, A. The Importance of the Electrochemical Environment in the Electro-Oxidation of Methanol on Pt(111). *ACS Catal.* **2016**, *6* (8), 5575–5586. https://doi.org/10.1021/acscatal.6b00931.

(6) Wang, H.-F.; Liu, Z.-P. Formic Acid Oxidation at Pt/H2O Interface from Periodic DFT Calculations Integrated with a Continuum Solvation Model. *J. Phys. Chem. C* **2009**, *113* (40), 17502–17508. https://doi.org/10.1021/jp9059888.

(7) Silbaugh, T. L.; Karp, E. M.; Campbell, C. T. Energetics of Formic Acid Conversion to Adsorbed Formates on Pt(111) by Transient Calorimetry. *J. Am. Chem. Soc.* **2014**, *136* (10), 3964–3971. https://doi.org/10.1021/ja412878u.

(8) Lović, J. D.; Tripković, A. V.; Gojković, S. Lj.; Popović, K. Dj.; Tripković, D. V.; Olszewski, P.; Kowal, A. Kinetic Study of Formic Acid Oxidation on Carbon-Supported Platinum Electrocatalyst. *J. Electroanal. Chem.* **2005**, *581* (2), 294–302. https://doi.org/10.1016/j.jelechem.2005.05.002.

(9) Perales-Rondón, J. V.; Herrero, E.; Feliu, J. M. Effects of the Anion Adsorption and PH on the Formic Acid Oxidation Reaction on Pt(111) Electrodes. *Electrochimica Acta* **2014**, *140*, 511–517. https://doi.org/10.1016/j.electacta.2014.06.057.

(10) Hansen, H. A.; Viswanathan, V.; Nørskov, J. K. Unifying Kinetic and Thermodynamic Analysis of 2 e– and 4 e– Reduction of Oxygen on Metal Surfaces. *J. Phys. Chem. C* **2014**, *118* (13), 6706–6718. https://doi.org/10.1021/jp4100608.

(11) Tripković, V.; Skúlason, E.; Siahrostami, S.; Nørskov, J. K.; Rossmeisl, J. The Oxygen Reduction Reaction Mechanism on Pt(111) from Density Functional Theory Calculations. *Electrochimica Acta* **2010**, *55* (27), 7975–7981. https://doi.org/10.1016/j.electacta.2010.02.056.

(12) Huang, J.; Zhang, J.; Eikerling, M. Unifying Theoretical Framework for Deciphering the Oxygen Reduction Reaction on Platinum. *Phys. Chem. Chem. Phys.* **2018**, *20* (17), 11776–11786. https://doi.org/10.1039/C8CP01315B.

(13) Gisbert, R.; García, G.; Koper, M. T. M. Adsorption of Phosphate Species on Poly-Oriented Pt and Pt(111) Electrodes over a Wide Range of PH. *Electrochimica Acta* **2010**, *55* (27), 7961–7968. https://doi.org/10.1016/j.electacta.2010.04.009.
(14) Li, N.; Lipkowski, J. Chronocoulometric Studies of Chloride Adsorption at the Pt(111) Electrode Surface. *J. Electroanal. Chem.* **2000**, *491* (1), 95–102. https://doi.org/10.1016/S0022-0728(00)00199-6.
(15) Hashiba, H.; Weng, L.-C.; Chen, Y.; Sato, H. K.; Yotsuhashi, S.; Xiang, C.; Weber, A. Z. Effects of Electrolyte Buffer Capacity on Surface Reactant Species and the Reaction Rate of CO2 in Electrochemical CO2 Reduction. *J. Phys. Chem. C* **2018**, *122* (7), 3719–3726. https://doi.org/10.1021/acs.jpcc.7b11316.
(16) Bui, J. C.; Lees, E. W.; Pant, L. M.; Zenyuk, I. V.; Bell, A. T.; Weber, A. Z. Continuum Modeling of Porous Electrodes for Electrochemical Synthesis. *Chem. Rev.* **2022**, *122* (12), 11022–11084. https://doi.org/10.1021/acs.chemrev.1c00901.
(17) Ringe, S.; Morales-Guio, C. G.; Chen, L. D.; Fields, M.; Jaramillo, T. F.; Hahn, C.; Chan, K. Double Layer Charging Driven Carbon Dioxide Adsorption Limits the Rate of Electrochemical Carbon Dioxide Reduction on Gold. *Nat. Commun.* **2020**, *11* (1), 33. https://doi.org/10.1038/s41467-019-13777-z.
(18) Wei, Z.; Zhang, M. K.; Zhu, B. Q.; Cai, J.; Chen, Y.-X. Mechanistic Insight into Formic Acid/Formate Oxidation at the Au(111) Electrode: Implications from the PH Effect and H/D Kinetic Isotope Effect. *J. Phys. Chem. C* **2022**, *126* (29), 11987–12002. https://doi.org/10.1021/acs.jpcc.2c03029.
(19) Bohra, D.; H. Chaudhry, J.; Burdyny, T.; A. Pidko, E.; A. Smith, W. Modeling the Electrical Double Layer to Understand the Reaction Environment in a CO 2 Electrocatalytic System. *Energy Environ. Sci.* **2019**, *12* (11), 3380–3389. https://doi.org/10.1039/C9EE02485A.
(20) R. Heil, S.; Holz, M.; M. Kastner, T.; Weingärtner, H. Self-Diffusion of the Perchlorate Ion in Aqueous Electrolyte Solutions Measured by 35 Cl NMR Spin–Echo Experiments. *J. Chem. Soc. Faraday Trans.* **1995**, *91* (12), 1877–1880. https://doi.org/10.1039/FT9959101877.
(21) Zhang, M.-K.; Wei, Z.; Chen, W.; Xu, M.-L.; Cai, J.; Chen, Y.-X. Bell Shape vs Volcano Shape PH Dependent Kinetics of the Electrochemical Oxidation of Formic Acid and Formate, Intrinsic Kinetics or Local PH Shift? *Electrochimica Acta* **2020**, *363*, 137160. https://doi.org/10.1016/j.electacta.2020.137160.
(22) Hochfilzer, D.; Xu, A.; Sørensen, J. E.; Needham, J. L.; Krempl, K.; Toudahl, K. K.; Kastlunger, G.; Chorkendorff, I.; Chan, K.; Kibsgaard, J. Transients in Electrochemical CO Reduction Explained by Mass Transport of Buffers. *ACS Catal.* **2022**, *12* (9), 5155–5161. https://doi.org/10.1021/acscatal.2c00412.
(23) Perales-Rondón, J. V.; Brimaud, S.; Solla-Gullón, J.; Herrero, E.; Jürgen Behm, R.; Feliu, J. M. Further Insights into the Formic Acid Oxidation Mechanism on Platinum: PH and Anion Adsorption Effects. *Electrochimica Acta* **2015**, *180*, 479–485. https://doi.org/10.1016/j.electacta.2015.08.155.
(24) Lamoureux, P. S.; Singh, A. R.; Chan, K. PH Effects on Hydrogen Evolution and Oxidation over Pt(111): Insights from First-Principles. *ACS Catal.* **2019**, *9* (7), 6194–6201. https://doi.org/10.1021/acscatal.9b00268.
(25) Scaranto, J.; Mavrikakis, M. HCOOH Decomposition on Pt(111): A DFT Study. *Surf. Sci.* **2016**, *648*, 201–211. https://doi.org/10.1016/j.susc.2015.09.023.

(26) Martínez-Hincapié, R.; Climent, V.; Feliu, J. M. Peroxodisulfate Reduction as a Probe to Interfacial Charge. *Electrochem. Commun.* **2018**, *88*, 43–46. https://doi.org/10.1016/j.elecom.2018.01.012.
(27) Wei, Y.; Zuo, X. Q.; He, Z. D.; Chen, W.; Lin, C. H.; Cai, J.; Sartin, M.; Chen, Y.-X. The Mechanisms of HCOOH/HCOO– Oxidation on Pt Electrodes: Implication from the PH Effect and H/D Kinetic Isotope Effect. *Electrochem. Commun.* **2017**, *81*, 1–4. https://doi.org/10.1016/j.elecom.2017.05.012.
(28) Betts, A.; Briega-Martos, V.; Cuesta, A.; Herrero, E. Adsorbed Formate Is the Last Common Intermediate in the Dual-Path Mechanism of the Electrooxidation of Formic Acid. *ACS Catal.* **2020**, *10* (15), 8120–8130. https://doi.org/10.1021/acscatal.0c00791.
(29) Huang, J. Surface Charging Behaviors of Electrocatalytic Interfaces with Partially Charged Chemisorbates. *Curr. Opin. Electrochem.* **2022**, 100938.
(30) Brimaud, S.; Solla-Gullón, J.; Weber, I.; Feliu, J. M.; Behm, R. J. Formic Acid Electrooxidation on Noble-Metal Electrodes: Role and Mechanistic Implications of PH, Surface Structure, and Anion Adsorption. *ChemElectroChem* **2014**, *1* (6), 1075–1083. https://doi.org/10.1002/celc.201400011.
